# Mutation D816V Alters the Internal Structure and Dynamics of c-KIT Receptor Cytoplasmic Region: Implications for Dimerization and Activation Mechanisms

**DOI:** 10.1371/journal.pcbi.1002068

**Published:** 2011-06-16

**Authors:** Elodie Laine, Isaure Chauvot de Beauchêne, David Perahia, Christian Auclair, Luba Tchertanov

**Affiliations:** LBPA, CNRS - ENS de Cachan, Cachan, France; University of California San Diego, United States of America

## Abstract

The type III receptor tyrosine kinase (RTK) KIT plays a crucial role in the transmission of cellular signals through phosphorylation events that are associated with a switching of the protein conformation between inactive and active states. D816V KIT mutation is associated with various pathologies including mastocytosis and cancers. D816V-mutated KIT is constitutively active, and resistant to treatment with the anti-cancer drug Imatinib. To elucidate the activating molecular mechanism of this mutation, we applied a multi-approach procedure combining molecular dynamics (MD) simulations, normal modes analysis (NMA) and binding site prediction. Multiple 50-ns MD simulations of wild-type KIT and its mutant D816V were recorded using the inactive auto-inhibited structure of the protein, characteristic of type III RTKs. Computed free energy differences enabled us to quantify the impact of D816V on protein stability in the inactive state. We evidenced a local structural alteration of the activation loop (A-loop) upon mutation, and a long-range structural re-organization of the juxta-membrane region (JMR) followed by a weakening of the interaction network with the kinase domain. A thorough normal mode analysis of several MD conformations led to a plausible molecular rationale to propose that JMR is able to depart its auto-inhibitory position more easily in the mutant than in wild-type KIT and is thus able to promote kinase mutant dimerization without the need for extra-cellular ligand binding. Pocket detection at the surface of NMA-displaced conformations finally revealed that detachment of JMR from the kinase domain in the mutant was sufficient to open an access to the catalytic and substrate binding sites.

## Author Summary

Protein kinases are involved in a huge amount of cellular processes through phosphorylation, a crucial mechanism in cell signaling, and their misregulation often results in disease. The deactivation of protein tyrosine kinases (PTKs) or their oncogenic activation arises from mutations which affect the protein primary structure and the configuration of the enzymatic site apparently by stabilizing the activation loop (A-loop) extended conformation. Particularly, mutation D816V of receptor tyrosine kinase (RTK) KIT, found in patients with pediatric mastocytosis, acute leukemia or germ cell tumors, can be considered as the archetype of mutation inducing a displacement of the population equilibrium toward the active conformation. We present a comprehensive computational study of the activating mechanism(s) of this mutation. Our multi-approach *in silico* procedure evidenced a local alteration of the A-loop structure, and a long-range structural re-organization of the juxta-membrane region (JMR) followed by a weakening of the interaction network with the kinase domain. Our results provided a plausible conception of how the observed departure of JMR from kinase domain in the mutant promotes kinase mutant dimerization without requiring extra-cellular ligand binding. The pocket profiles we obtained suggested putative allosteric binding sites that could be targeted by ligands/modulators that trap the mutated enzyme.

## Introduction

Regulation of physiological functions in the cell is mostly governed by phosphorylation – a crucial mechanism in cell signaling – catalyzed by protein kinases [1]–[5]. Stem cell factor (SCF) receptor or CD117, also known as human receptor tyrosine kinase (RTK) KIT (according to the nomenclature defined in [6]), belongs to the type III RTK family [7]–[10]. Type III RTKs consist of a glycosylated extra-cellular ligand-binding domain (ectodomain) connected to a cytoplasmic region by means of a single transmembrane helix. The cytoplasmic region of KIT is composed of an auto-inhibitory juxta-membrane region (JMR) and a protein tyrosine kinase (PTK) that is subdivided into proximal and distal lobes separated by an insert sequence of variable length (70–100 amino acids). In human KIT, the 77-amino acid kinase insert domain (KID) possesses phosphorylation sites and provides an interface for the recognition of pivotal signal transduction proteins [11]–[13].

**Figure 1:**
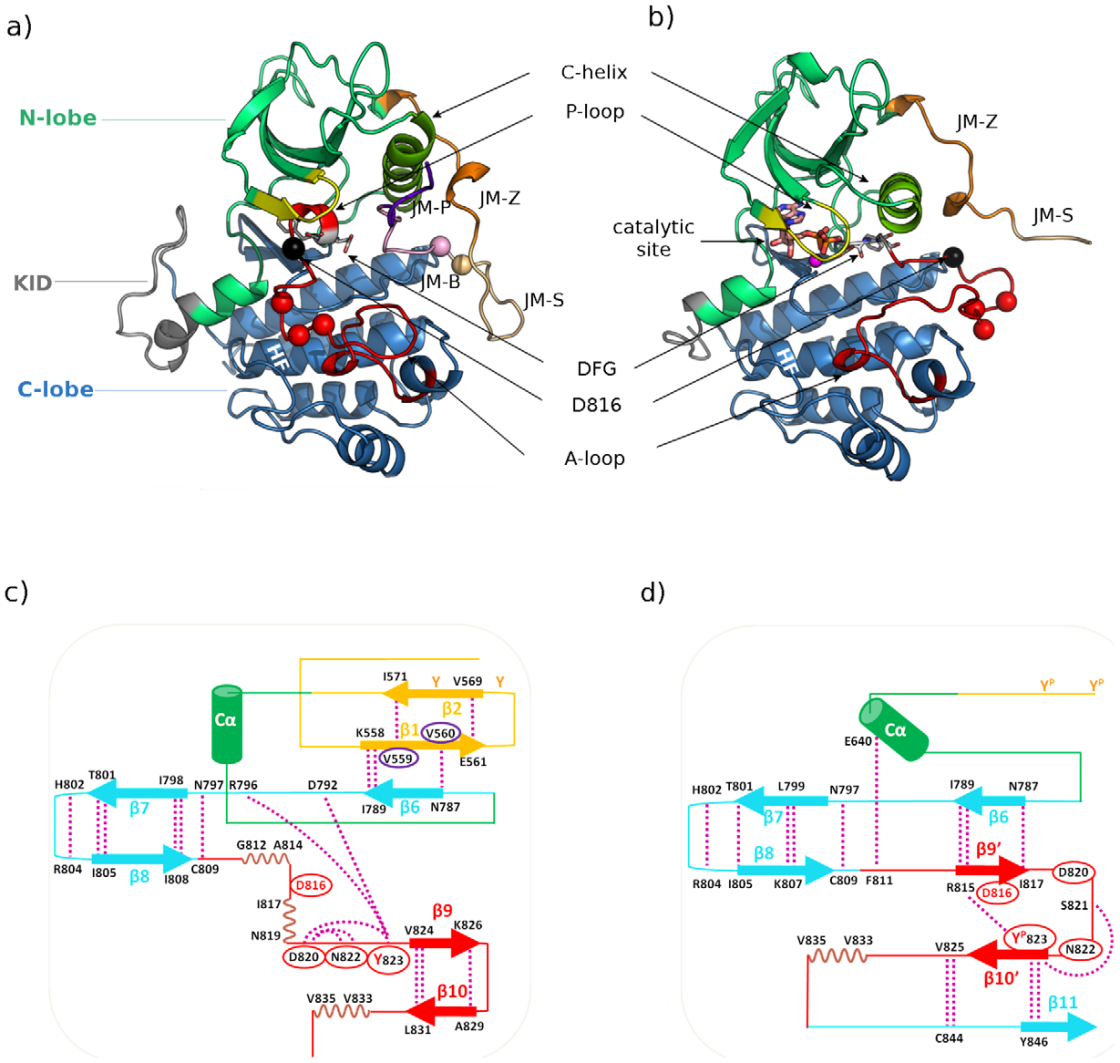
Structure of KIT cytoplasmic region in the inactive (auto-inhibited) and active states. ***Top***: Cartoon representation of wild-type KIT crystallographic structures: (**a**) inactive (auto-inhibited) state (PDB code 1T45) and (**b**) active state (PDB code 1PKG). The different domains composing the cytoplasmic region and key structural elements are labeled and highlighted in color. The N-terminal proximal lobe (N-lobe) is in green, the C-terminal distal lobe (C-lobe) is in blue and in-between the kinase insert domain (KID) is in grey. The segments JM-Proximal (JM-P, in purple), JM-Buried (JM-B, in pink), JM-Switch (JM-S, in light orange) and JM-Zipper (JM-Z, in orange) composing the juxta-membrane region (JMR) are specified. The activation loop (A-loop) is in red - its DFG motif highlighted in licorice with white carbons, the C-helix is in lime and the glycine-rich P-loop is in yellow. F-helix (HF) in the C-lobe is labeled in white. The position of the catalytic site is indicated in the active structure (**b**), where the bound ligand ADP is drawn with licorice and the Mg^2+^ ion is represented by a magenta sphere. The locations of mutational hot spot residues are indicated by spheres depicting the positions of their Cα atoms, with the D816V mutation highlighted in black. ***Bottom***: Schematic diagrams of the predicted secondary structure elements and interaction networks of JMR and A-loop: (**c**) inactive (auto-inhibited) state (1T45) and (**d**) active state (1PKG). JMR is in orange, N-lobe including C-helix (denoted Cα) is in green, C-lobe is in blue and A-loop is in red. Stabilizing H-bonds are drawn as dashed purple lines and the participating residues are labeled. Curved dashed purple lines represent turns. Phosphorylation sites Y568, Y570 and Y823 are highlighted. Encircled residues indicate mutational hot spots.

Binding of SCF to KIT leads to receptor dimerization [14], [15], intermolecular auto-phosphorylation of specific tyrosine residues [16] and PTK activation [8], [17], [18]. The activation process involves a large rearrangement of the activation loop (A-loop, ∼20–25 residues) situated in the C-lobe of PTK (**Figures 1a,b**). Conformational switch of A-loop from an inactive packed position (**Figure 1a**) to an active extended form (**Figure 1b**) releases access for Mg^2+^-ATP and protein substrate(s) to the kinase catalytic site [19], [20]. The inactive form of A-loop is maintained by JMR, which inserts directly in the domain interface between the N- and C-lobes of PTK (**Figure 1a**). JMR is composed of four fragments, namely JM-Proximal at the N-extremity (residues 547–552), the most buried JM-Binder (residues 553–559), JM-Switch (residues 560–570) and JM-Zipper (residues 571–581) [11], [21]. Phosphorylation of its primary sites Y568 and Y570 lifts the auto-inhibition (**Figure 1b**). Active KIT binds to intra-cellular substrates and phosphorylates them, thereby switching on multiple signaling pathways by interacting with enzymes and adaptor proteins [11], [22], [23]. For instance, the SCF-KIT interaction is essential for the development of melanocytes, erythrocytes, germ cells, mast cells and interstitial cells of Cajal (ICCs) [24]–[27].

The deactivation of tyrosine kinases or their oncogenic activation relates with mutations (point mutations as well as deletions and gene fusions) which affect the primary structure of the protein [28], [29]. A variety of mutations in the gene encoding the proto-oncogene KIT were found in different types of human cancer, in gastrointestinal stromal tumors (GISTs) [30], acute myeloid leukemia (AML) [31], mast cell leukemia (MCL) [32] and human germ cell tumors [33], among others. Mutations inducing tumorigenic effects were identified in the membrane-proximal Ig-like domain D5, in the auto-inhibitory juxtamembrane region and in the protein tyrosine kinase [34], [35]. Longley *et al.* [36] early proposed a classification of KIT gain-of-function mutations according to their structural and functional locations. The JMR mutations, frequently found in GISTs, are considered regulatory as they disrupt the auto-inhibitory mechanism which negatively regulates the activity of the protein [29], [37], [38]. The PTK mutations are considered catalytic as they directly affect the configuration of the enzymatic site probably by stabilizing the A-loop extended conformation [39]–[41]. To this category belongs the mutation of D in position 816 (indicated as a black sphere on **Figure 1 a, b**), most frequently substituted by V, found in most patients with mastocytosis, leukemia and germ cell tumors [42]. D816V is also resistant to Imatinib (Gleevec™) treatment [35], which has motivated to study its role in KIT activation mechanisms. Biochemical studies of KIT gave insights into the molecular mechanism of the ligand-independent activation of the D816V mutant receptor [43] but whether dimerization is required remains unclear [15], [44].

Crucial for kinase regulation is the orientation of the highly conserved Asp-Phe-Gly (D810-F811-G812) motif positioned at the N-extremity of A-loop, within the active site (**Figures 1a,b**). In the inactive state, the DFG triad adopts a “DFG-out” orientation for D810 points out of the ATP-binding pocket, while F811 is oriented toward the site and JMR is bound to PTK; in the active state, a canonical “DFG-in” conformation positions the catalytic D810 in the back of the site for chelation of magnesium while F811 is buried away and A-loop extends toward a completely solvent-exposed JMR. A-loop conformational switch is part of a global movement involving a tilt of the N-lobe towards the C-lobe and the rearrangement of several regions of the receptor such as the glycine-rich P-loop (residues 596–601, in yellow) and C-helix (residues 631–647, in lime) placed in the N-lobe (**Figure 1**). The link between the conformational changes of the DFG motif and a set of distinctive structural elements of the kinase core has recently been assessed through evolutionary analysis of PKs [45]. In addition, a surface comparison of PKs crystal structures has highlighted the role of F-helix (residues 766 to 786, labeled HF on **Figure 1**) in the C-lobe as a central scaffold for the dynamic assembly of the active kinase form [46].

The structural properties of PTKs were characterized mainly by X-ray analysis. Although crystallographic data yield valuable insights into such structural rearrangements, they represent only average conformation for a given set of crystallization conditions. Alternative experimental techniques, such as NMR spectroscopy, and computational approaches, such as molecular dynamics (MD) and normal mode analysis (NMA), provide a way to better understand the structure-dynamics-function relationships at the atomic level and further characterize the alteration of protein structure and internal dynamics induced by cancer mutations [47]. These theoretical methods also enable to describe intermediate conformational states, that can be used to guide the design of specific inhibitors acting as modulators of the enzymatic function by targeting putative allosteric sites [48], [49].

Recent (classical or advanced) molecular dynamics studies have begun to elucidate the molecular mechanisms of conformational transitions of PTKs [50]–[54] and to investigate the thermodynamic and mechanistic catalysts of kinase activation by cancer mutations [55]–[57]. Regarding drug-design oriented perspective, the inclusion of normal mode-based descriptions of protein flexibility was shown to improve the prediction of small molecules binding mode to PKs [58]–[61]. A combination of both methods, MD simulations and NMA (elastic network), was employed very recently to elucidate the inactive-to-active state transition of protein kinase B [62].

Early studies, including from our laboratory, shed light on the molecular mechanism by which the D816V mutation destabilizes A-loop inactive conformation, corresponding to a constitutive phosphotransferase activity [40], [63], [64]. This effect was then described in the context of Imatinib-induced resistance [65], [66]. The structure of KIT D816V mutant has not yet been determined. Nevertheless, recent crystallographic data [67] have suggested that the JMR auto-inhibitory conformation is destabilized in the D816H mutant, advocating a regulatory impact of this catalytic mutation. The mechanistic role of JMR in the initiation of the activation process was described by Zou *et al.* using classical and targeted MD [68]. The authors found that JMR is likely to detach from PTK before the A-loop conformational switch, due to electrostatic repulsion between the C-lobe and phosphorylated tyrosines in JMR.

In this study, we have carried out a detailed analysis of KIT receptor cytoplasmic region structural and dynamic changes related to D816V mutation through extensive description of the protein motions combining MD simulations and NMA. We first applied bioinformatics structural-based tools to accurately assign the secondary structure elements of JMR and A-loop and to characterize the hydrogen bonds stabilizing the active and inactive forms. We then employed homology modeling, MD simulations and free energy calculations to further evaluate the impact of the mutation on the stability of KIT cytoplasmic region auto-inhibited state. We observed both a local structural alteration and long-range structural and recognition effects on A-loop and JMR respectively, induced by the mutation. We then further explored the accessible motions of JMR relative to PTK with NMA and found that JMR is allowed larger amplitude motions in the mutant, promoting its triggering role for the inactive-to-active state transition. Displacements of MD conformations along chosen normal modes combined with pocket detection at the surface of the protein revealed that motions of JMR away from PTK in the mutant lead to the opening of an access to the catalytic and substrate binding sites. Consequently, we reveal D816V-induced alterations of KIT juxta-membrane region structure, dynamics and thermodynamics that were not previously described at an atomic level. We believe that our results may bridge experimental evidence of a regulatory activating role of the mutation and an alternative molecular recognition pattern determining the mutant dimerization.

## Results

### JMR and A-loop crucial interactions and stabilizing role

The crystallographic structures of KIT auto-inhibited inactive and active enzymatic forms (PDB codes: 1T45 [69] and 1PKG [40]) were carefully analyzed to correctly assign their secondary structure and characterize their stabilizing interactions (**Figures 1c,d**). In the inactive state (**Figure 1c**), JMR adopts a twisted hairpin (V-shaped) conformation, with the well-structured elements on the external part. The main predicted structural element is an anti-parallel β-sheet (β1–β2). The backbone of β1 (residues 558–561) interacts with the backbone of β2 (residues 569–571) and the backbone of β6 (residues 788–789) from the C-lobe of PTK, through strong and multiple H-bonds. A-loop inactive conformation shows a mixed structure composed of two 3_10_-helices (residues 812–814 and 817–819) adhered by a short coil and an anti-parallel β-sheet (β9–β10) stabilized by backbone-backbone H-bonds. In the active state (**Figure 1d**), JMR forms an extended coil that is fully solvent-exposed. A-loop is also positioned differently compared to the inactive state and displays a distinct secondary structure. Indeed the 810–820 sequence, featuring two helical motifs in the packed state, forms a β9' sheet (residues 815–816) separated from β10' (residues 823–824) by a turn in the extended conformation. The anti-parallel β-sheet β9'–β10' interacts with β6.

This structural-based analysis reveals that JMR and A-loop, two segments of KIT receptor cytoplasmic region capable of large conformational changes, exhibit distinct structural elements in the inactive and active forms of the enzyme, which are stabilized by peculiar hydrogen bonds. These elements are organized in such a way that residues from JMR and A-loop take the part of each other in the two structures interaction networks. The inactive packed conformation of A-loop is stabilized by intra-loop H-bond binding, whereas the active extended form is stabilized by interactions with the other regions of the receptor.

Noticeably, JMR and A-loop are the preferred regions of gain-of-function point mutations [11] (residues encircled in **Figures 1c,d**). The majority of these mutational hot spots participates in the stabilization of either the inactive (1T45) (**Figure 1c**) or active (1PKG) conformation (**Figure 1d**). For example, in the inactive form, V559 and V560 of β1 establish H-bonds with I571 of β2 and N787 of β6, respectively (**Figure 1c**). D816 serves as a negative capping for the 817–819 helix in 1T45 whereas it is a non-interacting residue of β9' in 1PKG, representing the active conformation (**Figure 1d**). D820 and N822 interact through their side-chains and participate in the formation of the 820–823 β-turn in the inactive state (**Figure 1c**). Y823 is involved in intra-loop interactions that stabilize turns in both states. Furthermore, this tyrosine is positioned in the catalytic site and H-bonded either to R787 or to the catalytic residue D792 in 1T45 (**Figure 1c**), whereas it bridges the anti-parallel β-sheet β10'–β11 by interacting with Y846 in 1PKG (**Figure 1d**). Its aromatic side chain was shown to be essential for the stabilization of the inactive conformation [70]. Mutation of any of these residues, except for V560, also confers resistance to Imatinib [35]. This analysis thus highlights the polymorphous structural properties of JMR and A-loop, tolerating mutations which provoke the deregulation of the kinase activity without altering the integrity of its structure.

### Impact of the D816V mutation on the inactive state conformational and thermodynamic stability

Four 50-ns MD simulations of full-length KIT receptor cytoplasmic region (CR), in wild-type (WT^547-935^, crystallographic) and D816V-mutated (MU^547-935^, modeled) forms, were run to explore and compare the protein internal dynamics and energetics. Two additional 50-ns simulations were carried out for KIT domains (WT^567-935^ and MU^567-935^), where residues 547–566 of JMR – disordered in the active structure 1PKG – were removed. Further we shall refer to these different forms of KIT CR as full-length (547–935) and truncated (567–935) and we shall identify the two MD simulations of WT^547-935^ by indices **1** and **2**, and the same for MU^547-935^.

**Figure 2:**
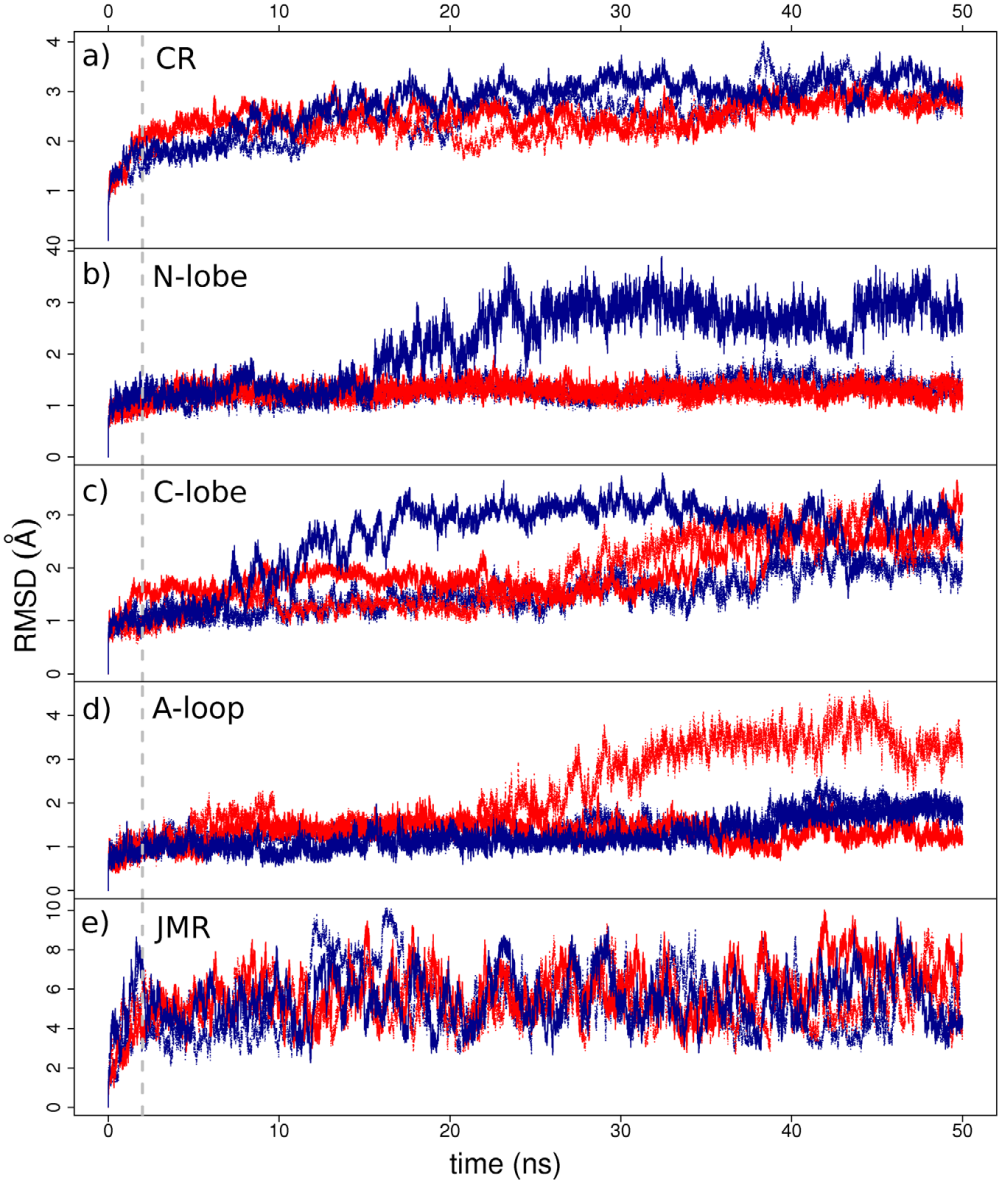
MD simulations of KIT cytoplasmic region in the auto-inhibited inactive form. The RMS deviations (in Å) were calculated from MD simulations of full-length CR for wild-type KIT, WT^547-935^ (in blue), and D816V mutant, MU^547-935^ (in red), on the backbone atoms of (**a**) the whole CR, (**b**) N-lobe, (**c**) C-lobe, (**d**) A-loop and (**e**) JMR. The 50-ns simulations **1** and **2** of the wild-type (mutant, respectively) are shown by plain and dashed lines. The dashed grey vertical line drawn at 2 ns indicates the relaxation time.

To analyze the global behavior of the studied systems, the root mean square deviations (RMSDs) of the nitrogen and carbon atoms of protein backbone with respect to the initial frame were plotted *versus* simulation time (**Figure 2a**). All four trajectories of full-length KIT CR, WT^547-935^ and MU^547-935^, display comparable backbone conformational drifts with RMSD mean values in the range 2.35–2.77±0.33–0.54 Å. Corresponding values for the truncated forms tend to be larger and vary much more, mean values of 3.39±0.74 Å and 3.59±1.05 Å for WT^567-935^ and MU^567-935^, respectively (**Figure S1a**). The evolution of RMSD during the course of the MD trajectories indicates a reasonable stability of the systems after a 2-ns relaxation period. Hence, the last 48 ns of each trajectory were considered as productive simulation time for further analyses. To find out which parts of the protein deviate most from the initial template, the RMSDs of backbone atoms were monitored for the N-lobe, C-lobe, A-loop and JMR, separately. The RMSDs of the N-lobe remained stable along all simulations except for simulation **1** of WT^547-935^ where it increased by 1.5 Å after 16 ns (**Figure 2b**). Also in this simulation, the RMSD of the C-lobe reached a stable level earlier than in the other runs (**Figure 2c**). The RMSD curves for N- and C-lobes of the truncated forms show comparable profiles to those of the full-length CRs (**Figures S1, **b–c****). A-loop displays a rather small deviation in both simulations of WT^547-935^ and in simulation **1** of MU^547-935^, with mean values in the range 1.26–1.43±0.25–0.35 Å, but the drift increased after 27 ns in simulation **2** of MU^547-935^ to reach a maximum of 4.58 Å (**Figure 2d**). The deviations of JMR are significantly larger than those of the other regions, with mean values in the range 5.26–5.90±1.07–1.65 Å, and their fluctuation profiles are unstable (**Figure 2e**).

The conformational stability of the studied systems was investigated through a convergence analysis of the trajectories [71]. Briefly, a set of *reference* structures are picked up randomly among the MD conformational ensemble and *reference* groups are formed, composed of conformations from the two halves of the trajectory (see **Materials and Methods**). A good convergence quality can be assessed when each *reference* structure is more or less equally represented in both halves of the trajectory. One defines a *lone reference* structure as a *reference* structure that is not represented in one half of the trajectory (one empty *reference* group) (insert in **Table 1**). To ensure the robustness of the method, the analysis was run with five different random seeds for the *reference* structure picking up (**Table 1**) similarly as we performed early [72]. *Lone reference* structures were found in almost all runs for the full-length forms, indicating a mean convergence quality for these simulations. The results were much improved for the truncated forms, especially regarding the wild-type. KIT wild-type cytoplasmic domain appears thus less stable in its full-length form than when the JMR is cleaved. The use of a 2.5 Å RMSD cutoff led to the identification of more reference structures in WT^547-935^ trajectories, indicative of a greater conformational diversity, than in MU^547-935^ trajectories (**Table 1**). By contrast, the optimal cutoff for WT^567-935^ (*r* = 3 Å) was smaller than that of MU^567-935^ (*r* = 3.5 Å). Consequently the D816V mutation seems to reduce the conformational variability of the full-length form but enhance that of the truncated form.

**Table 1:** Convergence analysis data on WT^547-935^, MU^547-935^, WT^567-935^ and MU^567-935^ MD trajectories.

Parameter	WT^547-935^		MU^547-935^		WT^567-935^	MU^567-935^
	1	2	1	2		
**Cutoff ** ***r*** ** (in Å)** *	2.5	2.5	2.5	2.5	3	3.5
***reference*** ** structures** **	4–7	5–7	2–4	4–6	3–4	3–5
***lone reference structures*** ***	5 (*1–4*)	5 (*2–4*)	3 (*0–2*)	5 (*1–2*)	1 (*0–1*)	3 (*0–2*)

*the RMSD cutoff *r* was chosen so as to best illustrate the conformational diversity of the MD ensembles;

**range of *reference* structures identified in the five runs;

***number of runs with *lone reference* structures together with the range of *lone reference* structures in the five runs (in italic).

**Figure 3:**
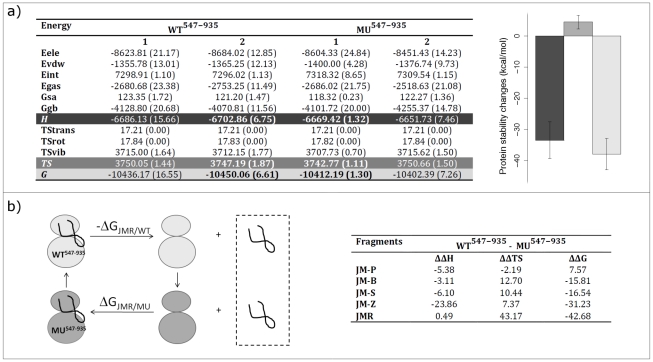
Protein stability and binding free energy changes between wild-type and D816V-mutated KIT in the inactive state. (**a**) Protein stability changes between wild-type and D816V-mutated KIT full-length CR: the table on the left gives the energetical contributions (in kcal/mol) computed on simulations **1** and **2** of full-length CR for wild-type (WT^547-935^) and mutant (MU^547-935^); the barplot on the right gives enthalpic (ΔH, in black), entropic (ΔTS, in dark grey) and total energy (ΔG, in light grey) changes computed from simulation **2** of WT^547-935^ and simulation **1** of MU^547-935^ (values in bold in the table). Statistical errors are given in parentheses in the table and represented as bars on the barplot. (**b**) Binding free energy changes of JMR and its fragments to KIT PTK between wild-type and mutant: the diagram on top represents a thermodynamic cycle picturing the dissociation of JMR from wild-type and mutated PTK; the table at the bottom gives the enthalpy (ΔΔH = ΔΔG_gas_+ΔΔG_gb_+ΔΔG_sa_), the entropy (ΔΔTS) and the total free energy (ΔΔG) differences (in kcal/mol), computed on the equilibrated conformations of WT^547-935^ and MU^547-935^. The different energetical contributions are defined in the **Materials and Methods** section.

The thermodynamic effect of the activating D816V mutation could be quantified by combining the equilibrium MD simulations with the Molecular Mechanics Generalized Born Surface Area (MM-GBSA) analysis [73] of KIT stability changes. Free energies were averaged over 2,400 conformations taken at 20-ps time interval along simulations **1** and **2** of WT^547-935^ and MU^547-935^ (**Figure 3a**). Errors on estimates were calculated using the method of Straatsma [74], useful for evaluating the uncertainty of finite correlated series [75], [76]. Predicted errors for the entropy components (TS_trans_, TS_rot_, TS_vib_, *TS*) were small in all simulations (up to 1.87 kcal/mol), reflecting very good convergence properties. Predicted errors for the enthalpic components (E_ele_, E_vdw_, E_int_, E_gas_, G_sa_, G_gb_, *H*) were larger (up to 24.84 kcal/mol). Nevertheless, error compensation occurred between large quantities and the auto-correlation functions suggested good convergence properties for the total enthalpy contribution *H* in simulation **2** of WT^547-935^ and simulation **1** of MU^547-935^. These two time series were thus retained to illustrate KIT enthalpic, entropic and total energy changes upon mutation on **Figure 3a**, although one should keep in mind that energy changes between WT^547-935^ and MU^547-935^ show the same trend whatever simulations considered.

We observed that the D816V mutation induced a significant decrease in the thermodynamic stability of KIT receptor cytoplasmic region autoinhibited inactive state (**Figure 3a**, on the right). This detrimental energetic effect was mainly due to loss of electrostatic interactions (E_ele_), whereas favorable van der Waals contributions were gained (E_vdw_) (**Figure 3a**, on the left). A slight reduction in conformational entropy (TS_tot_) was observed.

To qualitatively estimate the energetic impact of D816V mutation on KIT active versus inactive states, free energies were computed on the equilibrated inactive and active conformations of KIT truncated CR in wild-type and mutant forms (**Figure S2**, and **Materials and Methods**). We observed that the D816V mutation has a deleterious effect on both inactive and active conformations. However, the decrease in thermodynamic stability is smaller for the active form, so that the free energy difference between inactive and active conformations is reduced in the mutant compared to the wild-type.

Our free energy calculations thus indicate a deleterious impact of the D816V mutation on the stability of KIT receptor autoinhibited inactive state and enable to quantitatively relate the associated energy changes. They further suggest that the mutation may modify the energetic balance between inactive and active conformations. This finding is in excellent agreement with the data supporting a regulatory role for the D816V mutation [67] and correlates with similar results obtained for other kinases [55], [56].

### D816V-induced local structural alteration and long-range structural re-organization

**Figure 4:**
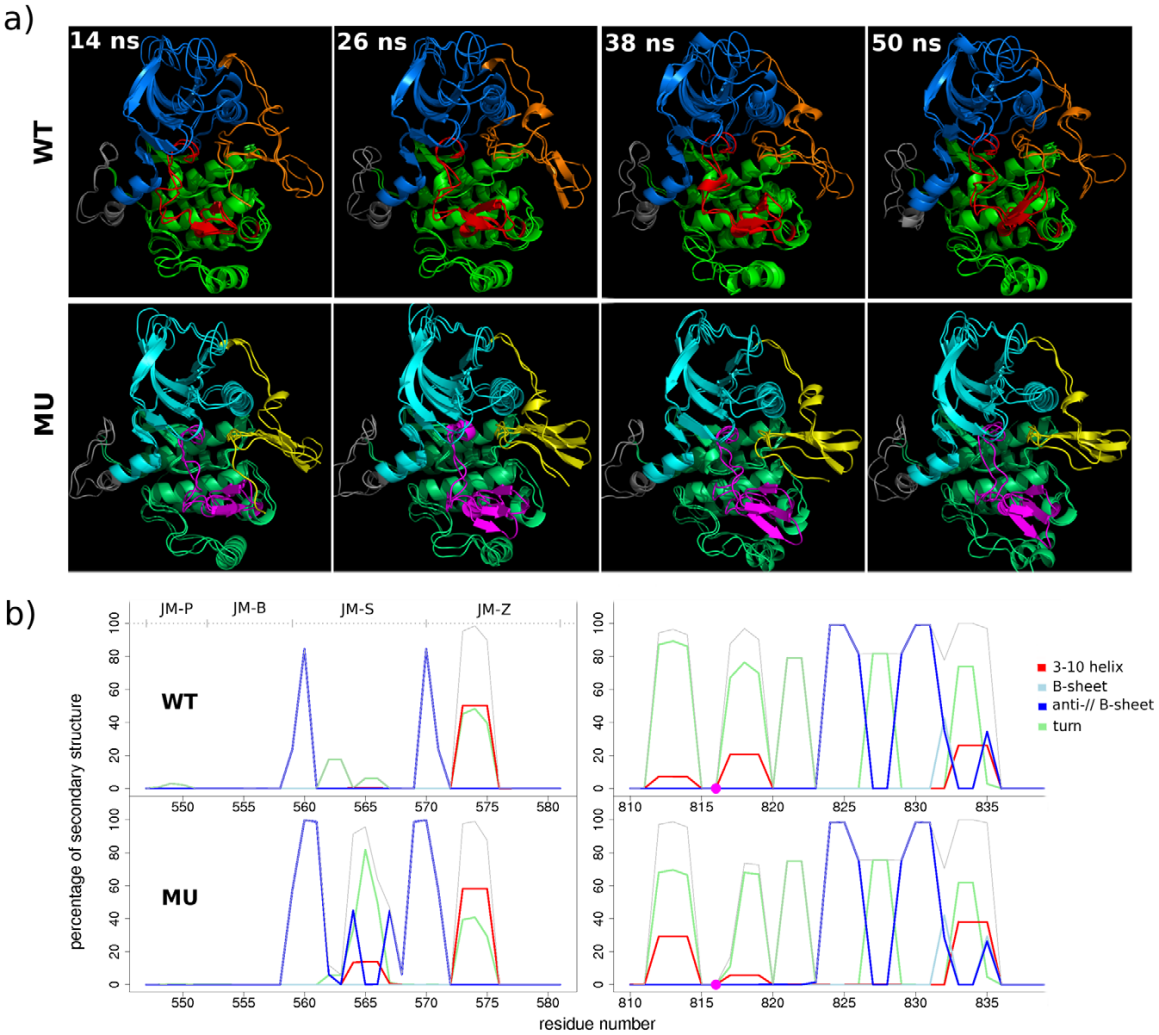
MD conformations and secondary structure of wild-type and D816V-mutated KIT cytoplasmic region in the inactive state. (**a**) MD conformations were taken at 14, 26, 38 and 50 ns from the two 50-ns MD simulations of wild-type KIT, WT^547-935^ (***upper panel***) and D816V mutant, MU^547-935^ (***lower panel***) and were superimposed by pair in cartoon representation. JMR is in orange (WT) and yellow (MU), N-lobe is in green (WT) and lime (MU), A-loop is in red (WT) and magenta (MU), and C-lobe is in marine (WT) and cyan (MU). (**b**) Secondary structure assignments for JMR (on the left) and A-loop (on the right) were averaged over the two 50-ns MD simulations of wild-type KIT, WT^547-935^ (at the top) and D816V mutant, MU^547-935^ (at the bottom). For each residue, the proportion of every secondary structure type is given as a percentage of the 96-ns total productive simulation time. 3_10_ helix is colored in red, parallel β-sheet in light blue, anti-parallel β-sheet in dark blue, turn in green and the cumulative sum in gray. The 816 position is indicated by a big point in magenta.

We analyzed the MD conformations in details to investigate the mutational effects of D816V on the internal structure and dynamics of KIT cytoplasmic region and understand what changes induced by this mutation promote the increased exchange rate between inactive and active states experimentally evidenced in [67]. MD snapshots taken at regular time intervals show that the A-loop position is systematically shifted between wild-type and mutated proteins (**Figure 4a**). For instance the small 817–819 helix, identified in our structural-based analysis of the inactive state is preserved in WT^547-935^ (**Figure 4a**, see in particular 38-ns snapshots) whereas it is unfolded in MU^547-935^. Secondary structure assignments averaged on MD trajectories confirm a 8% loss of helical structure (42% total structure loss) between residues 817 and 819 induced by the mutation (**Figure 4b**, right panel). This local destabilization results from the replacement of the negative capping D816 by a hydrophobic valine. Indeed H-bonds were recorded between the backbone and side-chain oxygen atoms of D816 and the backbone nitrogen atoms of K818, N819 and D820 for 22, 25 and 13% of WT^547-935^ total 96-ns productive simulation time whereas no H-bond was observed between V816 and residues 815–820 in MU^547-935^ simulations. Consistently, the computed solvent accessible surface area (SASA) of R815, mutated V816 and I817 were increased by 53, 31 and 84% in MU^547-935^ compared to WT^547-935^. The 817–819 helix unfolding was also assessed in the truncated form MU^567-935^. In addition, the helical contribution for residues 812–814 following the DFG motif is significantly larger (by 37%) in MU^547-935^ (**Figure 4a**, see in particular 26-ns snapshots) compared to WT^547-935^. This structural gain is counter-balanced by an equivalent decrease in the contribution of partially organized structure (turn) (**Figure 4b**, right panel). As a result, the proportion of helical/turn structures in this region are nearly identical in MU^547-935^, whereas it is displaced to the turn structure in WT^547-935^. The remaining part of A-loop (residues 820–835) shows a conserved structure between wild-type and mutated KIT. Consequently, we evidenced that the mutation D816V provokes an important alteration of the local structural organization of A-loop adjacent sequence regions.

**Figure 5:**
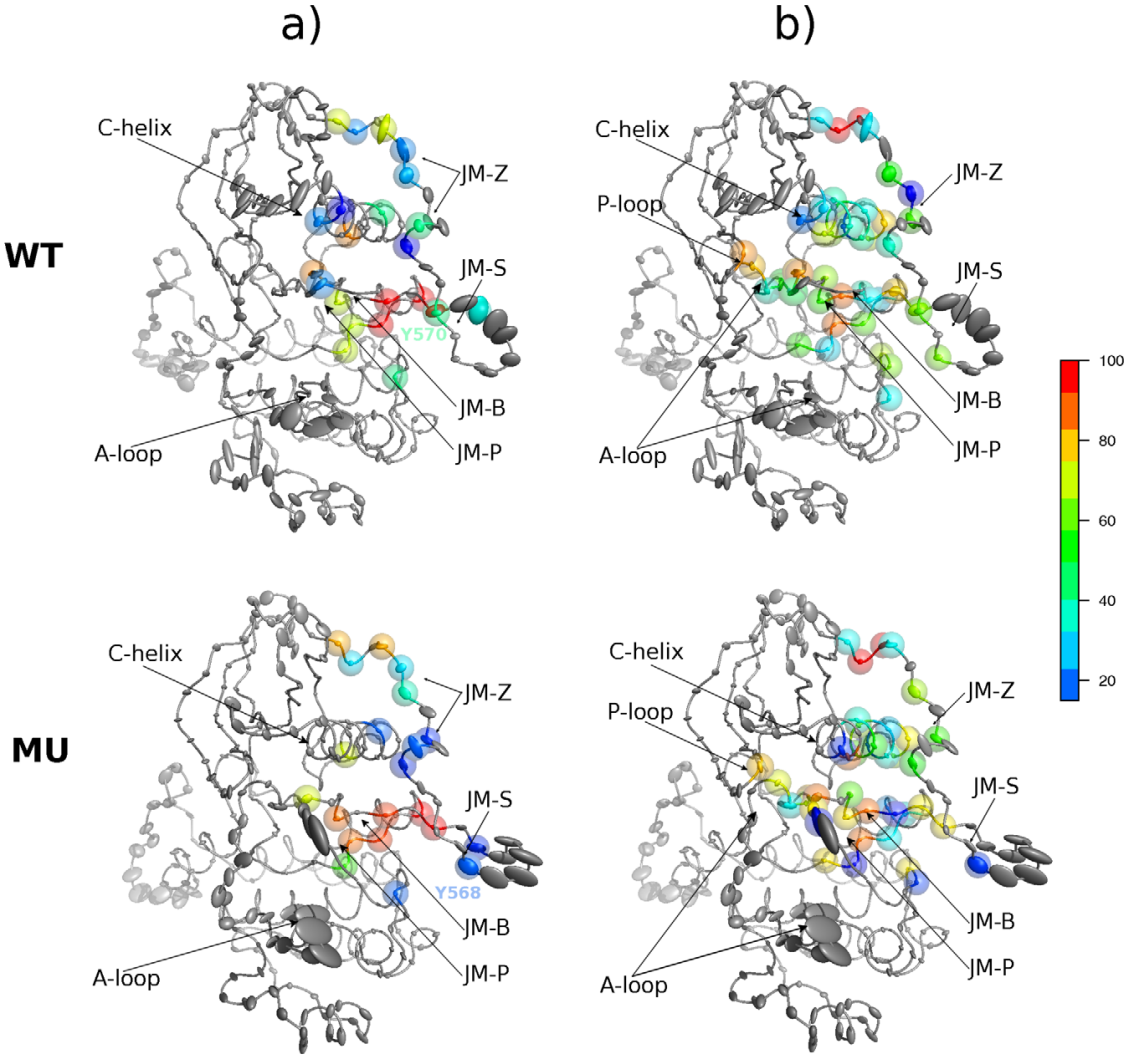
Interaction networks between JMR and PTK in auto-inhibited inactive KIT. **Upper Panel:** the wild-type, WT^547-935^. **Lower Panel:** the mutant, MU^547-935^. MD conformations averaged over the simulations **1** and **2** of WT^547-935^ and MU^547-935^ are represented as grey ribbons. Residues establishing at least one H-bond (**a**) or hydrophobic contact (**b**) between JMR and PTK for at least 15% of the total 96 ns productive simulation time are highlighted with colored transparent spheres. The color code, specified by the color scale bar on the right, goes from dark blue through green to red with increasing occupancy (in percentage of the simulation time). Ellipsoids drawn on Cα positions represent the anisotropic atomic fluctuations, computed after RMS fit onto the average MD conformation.

Apart from this local effect, we noted that the distantly positioned JMR rapidly adopted a well-shaped anti-parallel β-sheet structure in MU^547-935^, moving from a position packed to the C-lobe toward an axial position, whereas it retained its non well-ordered structure in WT^547-935^ (**Figure 4a**). Effective structural re-organization of residues 568–572 upon mutation can be assessed based on secondary structure assignment performed on the MD trajectories (**Figure 4b**, left panel). To clarify this observed long-range structural effect, the interaction network between JMR and PTK was characterized by recording H-bonds and hydrophobic contacts in WT^547-935^ (**Figure 5**, upper panel) and MU^547-935^ (**Figure 5**, lower panel). To describe in details JMR interaction network, we considered its fragments separately, JM-Proximal (JM-P), JM-Buried (JM-B), JM-Switch (JM-S) and JM-Zipper (JM-Z). To visualize the established contacts occupancy, we used a color gradient from red to blue for strong to weak interactions, respectively. Upon mutation, both types of interactions, H-bonds and hydrophobic, vanish between JM-P/JM-B (residues 550–553) and the N-extremity of C-helix (residues 632–633) (**Figure 5**). H-bonds between JM-Z (residues 573–576) and C-helix (residues 640–642) (**Figure 5a**) and hydrophobic contacts between JM-P/JM-B (residues 551–553) and both P-loop and A-loop (**Figure 5b**) are weakened in the mutant. Noticeably, in the mutant JM-S also interacts less strongly with the C-lobe, including with β6. The two primary phosphorylation sites (JM-S) apparently swap their role in the interaction network between wild-type and mutated forms. Indeed Y846 in the C-lobe establishes a H-bond either with Y570 (46%) in WT^547-935^ or with Y568 (27%) in MU^547-935^ (**Figure 5a**). Moreover a hydrophobic contact between Y570 and I789 (β6) persistently exists in WT^547-935^ (55%), while it is not observed in MU^547-935^. Consequently, the long-range effect of the D816V mutation leading to noticeable structural re-organization and shifted position of JMR, is accompanied by an alteration of the interaction network between JMR and PTK.

The Cα atomic fluctuations depicted by ellipsoids on the averaged MD conformations (**Figure 5**) show two highly flexible clusters, with fluctuations between 2.4 and 4.5 Å corresponding to JM-S and A-loop in both wild-type (residues 563–567 and 827–829) and mutant (residues 563–567, 817, and 825–830). By contrast, JM-Z (residues 581–582), the loop preceding C-helix (residues 630–632) and the loop preceding G-helix in the C-lobe (residues 871–873 and 877–878) display high flexibility with values above 2.4 Å up to 3.6 Å in WT^547-935^ (**Figure 5**, upper panel), whereas their fluctuations are much reduced in MU^547-935^ (**Figure 5**, lower panel).

**Figure 6:**
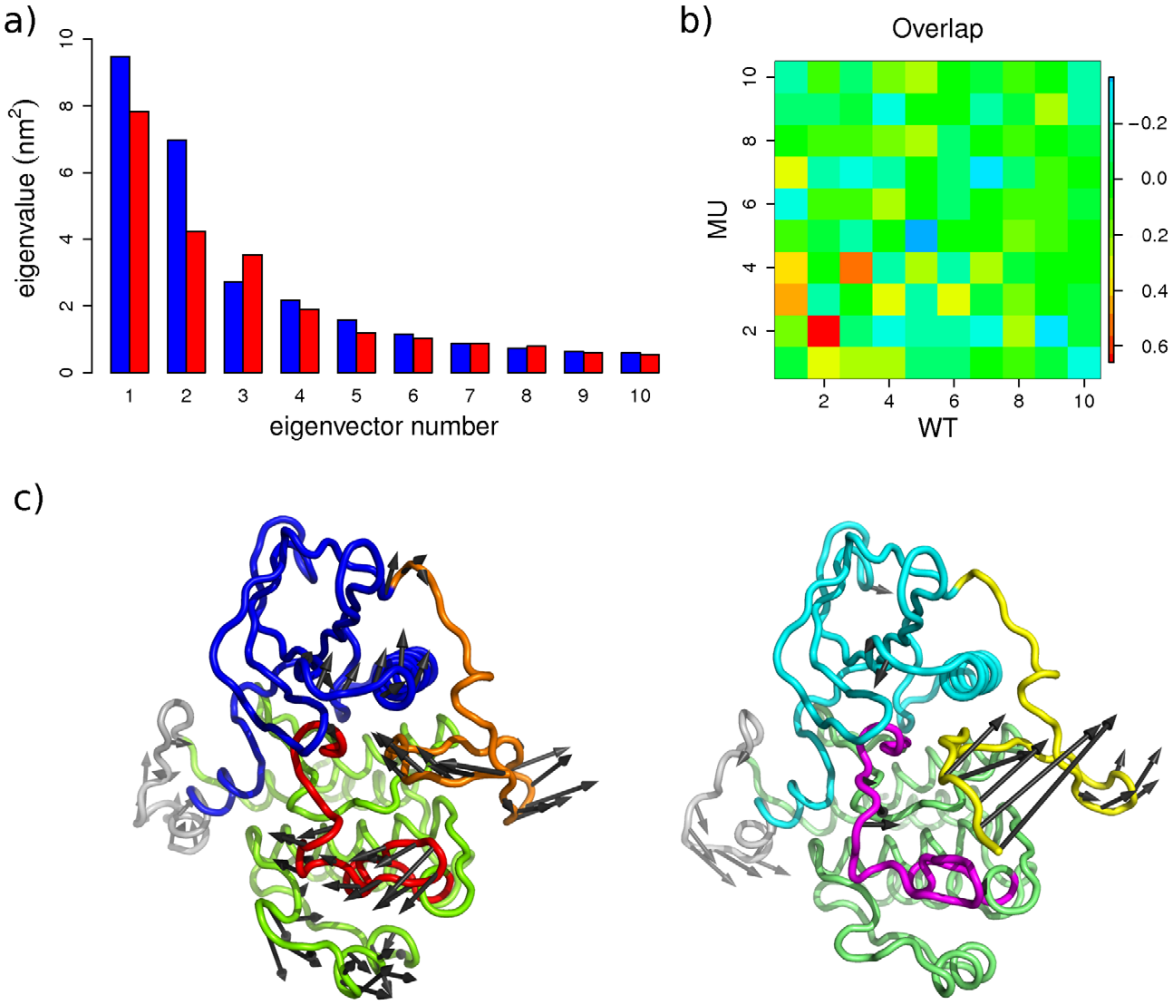
Principal component analysis of KIT cytoplasmic region motions in the auto-inhibited inactive form. The calculation was performed on the backbone atoms of WT^547-935^ and MU^547-935^, combining the MD trajectories **1** and **2** (total of 96-ns productive simulation time) and taking the average MD conformations as references for the RMS fits. ***Top***: (**a**) The barplot gives the eigenvalues spectra of wild-type (in blue) and mutant (in red), in descending order. (**b**) The grid gives the overlaps between eigenvectors from wild-type (columns) and mutant (rows). The overlap or similarity between two eigenvectors is evaluated as their scalar product and represented by a colored rectangle, from blue (−0.7) though green (0) to red (0.7). ***Bottom***: (**c**) Modes 2 atomic components for wild-type (left panel) and mutant (right panel) are drawn as dark grey arrows on the protein cartoon representation. JMR is in orange (WT) and yellow (MU), N-lobe is in green (WT) and lime (MU), A-loop is in red (WT) and magenta (MU), C-lobe is in marine (WT) and cyan (MU).

To further characterize KIT receptor cytoplasmic region motions in the inactive state, principal component analysis (PCA) of the MD trajectories was performed. 26 and 28 PCA modes are sufficient to describe 90% of the total backbone fluctuations of WT^547-935^ and MU^547-935^, respectively. The first three PCA modes cumulative contribution is 56% for the wild-type and 53% for the mutant (**Figure 6a**). Computed scalar products between the first ten PCA modes from the two proteins indicate that the correspondence is not straightforward between the two ensembles. However, the second principal modes share a high degree of 66% similarity (**Figure 6b**). Noticeably, mode 2 of WT^547-935^ bears a significantly larger contribution (20%) than mode 2 of MU^547-935^ (14%) (**Figure 6a**), and displays a two-fold higher degree of collectivity *k* (see **Materials and Methods**). Indeed, it illustrates atomic motions of JMR coupled to deformations of PTK in the N-lobe – interface with JM-Z and residues 627–633 preceding C-helix, and in the C-lobe – A-loop and residues 868–886 including G-helix (**Figure 6c**, left panel), whereas mode 2 of MU^547-935^ describes atomic motions of JMR independent from PTK (**Figure 6c**, right panel).

Consequently, the D816V mutation alters the global dynamics of KIT receptor cytoplasmic region, in particular regarding the participation of JMR in the main motions of the protein. The coupling between JMR and the N-lobe revealed by the PCA in wild-type KIT can be related to the large atomic fluctuations observed in the N-lobe of this form. By contrast in the mutant, no coupling is observed and fluctuations in the N-lobe are much smaller. These findings are also in agreement with the greater conformational variability of the wild-type over the mutant evidenced by the convergence analysis.

PCA applied on the MD trajectories of the truncated proteins (WT^567-935^ and MU^567-935^) reveals that independent motions of the residues 567–581 of the JMR portion (JM-Z and part of JM-S) are dominant in the total backbone fluctuations of the protein, contributing up to 52% and 74%, respectively. Indeed, the reduced JMR is highly solvent-exposed and flexible. It displays larger fluctuations in MU^567-935^ than in WT^567-935^ (**Figure S3**) and thus appears to be responsible for the greater conformational variability of the cleaved mutant over the cleaved wild-type evidenced by the convergence analysis. Noticeably, the A-loop is not further destabilized in these cleaved forms and its positions at the end of the simulations superimpose well between WT^547-935^ and WT^567-935^ on the one hand, MU^547-935^ and MU^567-935^ on the other hand (**Figure S3**). This observation is consistent with a recent biochemical characterization of KIT cytoplasmic domain showing that the cleavage of JMR does not automatically promote inactive-to-active transition of the A-loop [70].

### JMR triggering role for the inactive-to-active state transition promoted by the mutation

To elucidate the thermodynamic origin of the structural and dynamics changes induced by the mutation in the remote JMR, we evaluated binding free energy changes between the equilibrated conformations of wild-type and mutated KIT (**Figures 3c**), where no structural re-organization of JMR was yet observed. Single-point MM-GBSA calculations were performed following the thermodynamic cycle shown on top of **Figure 3c** to estimate the relative attachment of JMR to PTK. We found a global binding free energy change (ΔΔG) of −42.68 kcal/mol, indicating that JMR is more tightly attached to PTK in the wild-type than in the mutant, due to a largely more favorable (mainly caused by the vibrational component) entropy (**Figure 3c**, table at the bottom). The greater conformational variability displayed by WT^547-935^ compared to MU^547-935^ in the simulations may enlighten this entropic effect. Indeed, the penalty endorsed by JMR and PTK upon binding due to reduction of their intrinsic vibrational entropy may be balanced in the wild-type by an emerging cooperativity between the two domains, as suggested by the PCA. By contrast in the mutant, the absence of cooperativity may lead to a larger entropic penalty upon binding. Binding free energy changes computed for the different JMR fragments show a dominant entropic penalty for JM-B and JM-S binding to MU^547-935^ compared to WT^547-935^. The largest energy change is observed for JM-Z – covalently bound to PTK, due to both large enthalpic and entropic penalties in mutated KIT. The smallest energy change is obtained for the solvent-exposed extremity JM-P. Overall, these calculations reveal the thermodynamic determinants responsible for the alteration of JMR structure and dynamics upon mutation and they enable to formulate the hypothesis that JMR is less tightly attached to PTK in the mutant than in the wild-type.

To further explore the motions accessible to JMR, all-atom Normal Mode Analysis (NMA) was conducted on representative MD conformations. Based on our convergence quality assessment (**Table 1**), we retained simulations **1** of WT^547-935^ and MU^547-935^ as they displayed the smallest number of *lone reference* structures. Two sets of four MD conformations – extracted through clustering analysis (see **Materials and Methods**), were considered, taken at: 4217, 34238, 42356, 49260 ps for WT^547-935^ and 2531, 19157, 30160, 36987 ps for MU^547-935^. These sets enabled to get the best convergence quality and thus were the most representative of the MD conformational ensemble. For comparison, NMA was also performed on the static crystallographic structure 1T45 [69]. The 97 non-zero lowest-frequency modes (ω<20 cm^−1^) obtained from each NMA were considered, leading to a total of 97 values for the X-ray structure and 388 values for the wild-type and the mutant respectively. On this ensemble, the degrees of collectivity of JMR atomic motions, 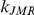, were computed, with values ranging from 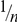 (only one atom among the total *n* involved in the motion) to 1 (high collectivity). The mean 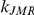 value is 0.57 for WT^547-935^ and 0.60 for MU^547-935^, with 48% and 54% of the values above 0.6 respectively (**Figure S4**). This indicates that JMR atomic motions illustrated by the normal mode ensemble are overall more collective in the mutant than in the wild-type. For comparison, a significantly lower mean value 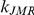 = 0.51 is found for the X-ray structure, underlining the relaxation of the protein in the MD simulations.

**Figure 7:**
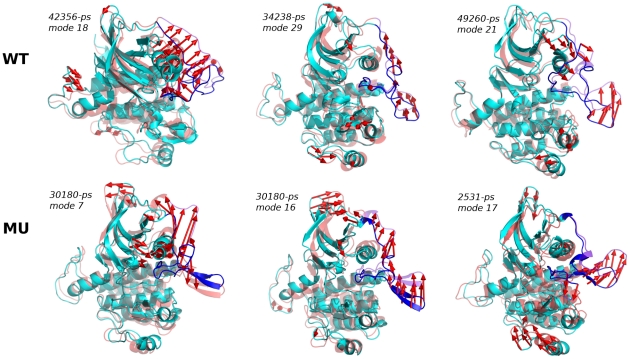
Normal modes illustrating atomic motions accessible to JMR in KIT auto-inhibited inactive form. ***Upper Panel*** **:** the wild-type, WT^547-935^. ***Lower Panel*** **:** the mutant, MU^547-935^. Each mode is labeled and the MD conformation from which it was obtained is given. The two extreme conformations along each mode in each direction are displayed in cartoon representation, in opaque cyan (JMR in blue) and transparent red (JMR in purple). The atomic (Cα) components of each mode are drawn as red arrows.

All calculated normal modes represent a limited ensemble of motions, as several modes significantly overlap. We describe qualitatively some of the modes which exhibit motions of JMR relatively to PTK. Three modes from WT^547-935^ and three modes from MU^547-935^ were picked up for their displaying of JM-Z and/or JM-S large displacements (**Figure 7** and **Table 2**). In WT^547-935^, mode 18_{42356-ps}_ shows a collective motion of JMR of especially large amplitude for JM-Z (**Table 2**), coupled to a rigid-body motion of C-helix (**Figure 7**, upper left panel). In MU^547-935^, mode 7_{30180-ps}_ displays an even larger resultant displacement of JM-Z but a rather low 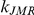 (**Table 2**), indicative of disparities in the mode atomic components (**Figure 7**, lower left panel). Large displacements of JM-S are displayed in modes 21_{49260-ps}_ of WT^547-935^ and 17_{2531-ps}_ of MU^547-935^ (**Table 2**). JM-S concerted atomic motions are rather coupled to JM-P and the loop preceding C-helix in mode 21_{49260-ps}_ of the wild-type (**Figure 7**, upper right panel) and to JM-P and G-helix in mode 17_{2531-ps}_ of the mutant (**Figure 7**, lower right panel). Modes 29_{34238-ps}_ of WT^547-935^ and 16_{30180-ps}_ of MU^547-935^ illustrate combined displacements of JM-Z and JM-S with 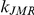 above 0.6 (**Table 2**). Concerted JMR atomic motions are oriented toward the back of PTK in mode 29_{34238-ps}_ of the wild-type (**Figure 7**, upper middle panel) while the arrows representing JMR atomic motions in mode 16_{30180-ps}_ of the mutant point away from PTK and are more numerous (**Figure 7**, lower middle panel), corresponding to a very high degree of collectivity 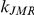 (**Table 2**).

**Table 2:** Characteristics of six normal modes representative of WT^547-935^ and MU^547-935^ inactive KIT motions.

KIT	Mode_{conformation}_ *	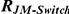 **	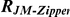 **	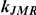 ***
**WT^547-935^**	18_{42356-ps}_	0.50 (60^th^)	0.79 (4^th^)	0.64
	29_{34238-ps}_	0.62 (22^nd^)	0.75 (5^th^)	0.68
	21_{49260-ps}_	0.81 (3^rd^)	0.42 (90^th^)	0.62
**MU^547-935^**	7_{30180-ps}_	0.50 (80^th^)	1.00 (1^st^)	0.31
	16_{30180-ps}_	0.81 (9^th^)	0.63 (11^th^)	0.84
	17_{2531-ps}_	0.90 (3^rd^)	0.30 (180^th^)	0.56

*Each mode is identified by its index along with the simulation time corresponding to the MD conformation (in ps) on which the NMA was applied;

**
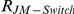
and 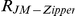 are the norms of the resultant displacement vectors of JMS and JMZ respectively along the mode;

***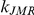 is the degree of collectivity of the mode calculated on the atoms of JMR.

The selected normal modes reveal differences in the motions accessible to JMR in wild-type and mutated KIT. Consistent with the PCA results, the JMR atomic displacements in the wild-type are coupled to deformations in PTK, in particular to motions of C-helix and its preceding loop; in the mutant, the JMR atomic motions are more independent from PTK. In particular, mode 16_{30180-ps}_ represents a possible way-out of JMR from PTK through a highly collective motion.

**Figure 8:**
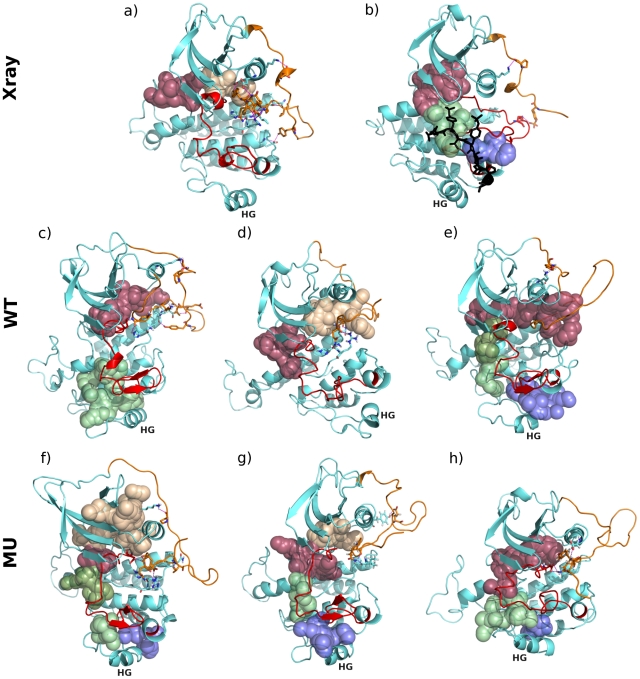
Pockets detected on the surface of wild-type and D816V-mutated KIT cytoplasmic region. **Upper Panel:** X-ray structures of KIT (**a**) in the inactive (auto-inhibited) form (1T45) and (**b**) in the active form (1PKG, chain A). **Middle Panel:** Conformations obtained from the 4-Å displacement of wild-type KIT along normal modes (**d**) 18_{42356-ps}_, (**c**) 29_{34238-ps}_ and (**e**) 21_{49260-ps}_. **Lower Panel:** Conformations obtained from the 4-Å displacement of D816V mutant along normal modes (**f**) 7_{30180-ps}_, (**g**) 16_{30180-ps}_ and (**h**) 17_{2531-ps}_. The core of the protein is in cyan, JMR is in orange, A-loop is in red and G-helix, which serves as a platform for the substrate in 1PKG, in labeled in black. The substrate is displayed in black cartoon and licorice on 1PKG structure (**b**). Residues involved in H-bonds between JMR and the PTK are drawn with sticks and the H-bonds are indicated by dashed purple lines. Pockets are displayed in red when they overlap with the catalytic site, in orange when they are located between the catalytic site and JMR, in olive, green or purple when they overlap with the substrate binding site.

The wild-type and mutated structures were displaced up to 4 Å with a step of 0.1 Å along each selected normal mode in both positive and negative directions (**Figure S5**). The extreme conformations obtained from the 4-Å displacements of JMR away from PTK enable to produce JMR atomic motions (**Figures 8c–h**). Based on visual inspection of the modes components, C-helix appears more attached to JMR in wild-type conformations, resulting in deformations of PTK, than in mutated conformations (**Figure 8**). Furthermore, some extreme conformations show a coil structure of A-loop in the wild-type and mutant (**Figure 8d, h**).

A close inspection of the surface of wild-type and mutated proteins permitted to identify pockets on the NMA-displaced conformations and on the crystallographic structures 1T45 and 1PKG (**Figure 8a,b**). According to X-ray data, in the inactive state the extended catalytic region shows two adjacent pockets (**Figure 8a**, the areas colored in red and orange); while in the active state, we observed the occurrence of three adjacent pockets form the ATP-binding site and the substrate binding site (**Figure 8b**, the areas colored in red, green and purple, respectively). These three pockets are also detected in some extreme conformations obtained from the 4-Å displacement in mutant (**Figure 8g and h**). A more fragmented pocket profile is observed in conformations (f) of the mutant and (e) of the wild-type, with an additional small pocket (in olive). Consequently, pocket search applied to conformations obtained from NMA reveals that displacing JMR relative to PTK leads to the opening of a “path” to the catalytic site, even though A-loop remains in its inactive state. The access to the catalytic site is particularly facilitated in mutated KIT, whose structure (g) displaced along mode 16_{30180-ps}_ displays a “path-of-pockets” very similar to that observed in X-ray structure 1PKG, in terms of volumes and shapes. Consistently, pocket search performed at the surface of the 50-ns MD conformations of WT^567-935^ and MU^567-935^ revealed a similar “path-of-pockets” in the mutant truncated CR (**Figure S6**). Overall these thorough normal mode analyses suggest that the D816V mutation may promote spontaneous detachment of JMR from PTK and concomitant access release to the substrate and ATP-binding sites, hence reinforcing the triggering role of JMR in the inactive-to-active state transition of the protein.

## Discussion

This study represents a detailed description at the atomic level of the impact of the D816V mutation on KIT cytoplasmic region structure, internal dynamics and thermodynamic stability and contributes to the basic concepts of the activating/deactivating mechanisms of RTKs. Unlike for many other kinases, the activation of type III RTKs such as KIT does not require the phosphorylation of the activation loop [70]. Instead, the primary phosphorylation sites are located in the juxta-membrane region, whose detachment from its auto-inhibitory position is likely to trigger the inactive-to-active state transition, due to repulsive negative charges [68]. Our structural-based bioinformatics analysis of KIT receptor auto-inhibited inactive and active states highlighted the strong polymorphous character of both A-loop and JMR and their crucial stabilizing roles for either conformation. We also found that mutational hot spots located in these two elements of the receptor play important role in the secondary structure stabilization and H-bond patterns of the protein. In particular, residue D816 in A-loop serves as a negative capping for a helical motif in the inactive form whereas it stands within a β-sheet in the active form.

Inspired by these preliminary observations, we were interested in characterizing the effect of the mutation on the auto-inhibition mechanism of KIT receptor cytoplasmic region. Hence we chose to explore the conformational space around KIT auto-inhibited inactive state, in wild-type and mutated forms, using multiple 50-ns MD simulations. The striking similarity between wild-type KIT (1T45 [69]) and the mutant D816H (3G0F [67]) suggested that the folding of the protein would be similar in the context of the mutant D816V.

In our simulations, A-loop demonstrated high flexibility, consistently with NMR studies [77]. We also evidenced a local structural alteration induced by D816V on A-loop inactive conformation. In particular, we observed unfolding of the small 817–819 3_10_ helix in the mutant. As we pointed out in the introduction, this local effect has been previously characterized [40], [63], [64], [67]. By contrast, the behavior of the juxta-membrane region had not yet been explored in the context of the mutant. Our simulations revealed a long-range structural re-organization of JMR and a conformational drift of JM-Switch segment, from a position packed to the C-lobe to an axial arrangement, in the mutated form. Comparing our data with those obtained during the first third of a targeted MD simulation of the inactive-to-active state transition of wild-type KIT cytoplasmic region [68]), we propose that the drift we observed could be the first step towards the ligand-independent activation of D816V mutant.

Our recording of the hydrogen bonds and hydrophobic contacts gave a possible justification for this long-range effect, through the weakening of the interaction network between JMR and both N-lobe and C-lobe of PTK upon mutation. Furthermore, quasi-harmonic analysis (PCA) of the trajectories and computed free energy changes revealed that the mutation has a deleterious impact on the thermodynamic stability of the inactive state and on the coupling between JMR and catalytic domain. In the literature, differences between homology models of active wild-type KIT receptor kinase domain and the mutant D816V early suggested a strong influence of JMR on the folding of wild-type PTK but not on that of D816V-mutated PTK [64]. Moreover, it was reported recently that mutation in position 816 shifts the conformational equilibrium of the kinase away from the auto-inhibited state toward JMR being released to solvent and disordered [67]. These *in silico* and *in vitro* results are in good agreement with the coupling/decoupling balance put in light here between the wild-type and mutated proteins.

In an attempt to go beyond crystallographic structures or homology models static view and to get a qualitative insight into the modification of the protein energetic landscape upon mutation, we have conducted normal mode analysis on *representative* conformations sampled in our MD simulations. This method is inscribed in the same philosophy as consensus normal modes theoretical framework [78]. Including the well equilibrated first hydration shell from MD simulations permitted to obtain modes with good accuracy regarding JMR, which is located at the surface of the protein. Statistics computed on the NMA ensembles revealed more collective motions of JMR in the mutant. On one side, the overlap between different starting points reflects conformation population equilibrium; on the other side, the identification of particular modes for particular conformations relates to the search for a discrete transition path between inactive and active states. In this regard, several modes were chosen that described possible ways-out for JMR to depart from catalytic domain. A pocket search at the surface of the conformations displaced along these modes revealed that JMR accessible displacements relative to mutant PTK were sufficient to open a *path* of adjacent pockets to the substrate-binding sites. This observation was also confirmed by the MD simulations of the truncated proteins, where JMR was cleaved.

Noticeably, proto-oncogenic mutations located downstream of the DFG motif in the A-loop, as is the case of D816V, were identified in at least seven other kinases, including BRAF (V600) and EGFR (L858/L861) [79]. MD studies of EGFR evidenced a deleterious impact of mutation L858R on the thermodynamic stability of the protein inactive state [55], [79], the effect described here for KIT mutation D816V. It was also revealed that conformational changes in L858R and L861Q EGFR mutant, taking place in the A-loop and C-helix, may facilitate the inactive-to-active state transition. Regarding BRAF, Xie et al. proposed a mechanism by which mutation V600E mimics the effect of phosphorylation events in the A-loop, thus disrupting the kinase inactive conformation toward the active state [80]. These results match with our observations, according to which KIT mutation D816V favors departure of JMR from PTK, a process that is normally induced by phosphorylation events in the wild-type protein.

**Figure 9:**
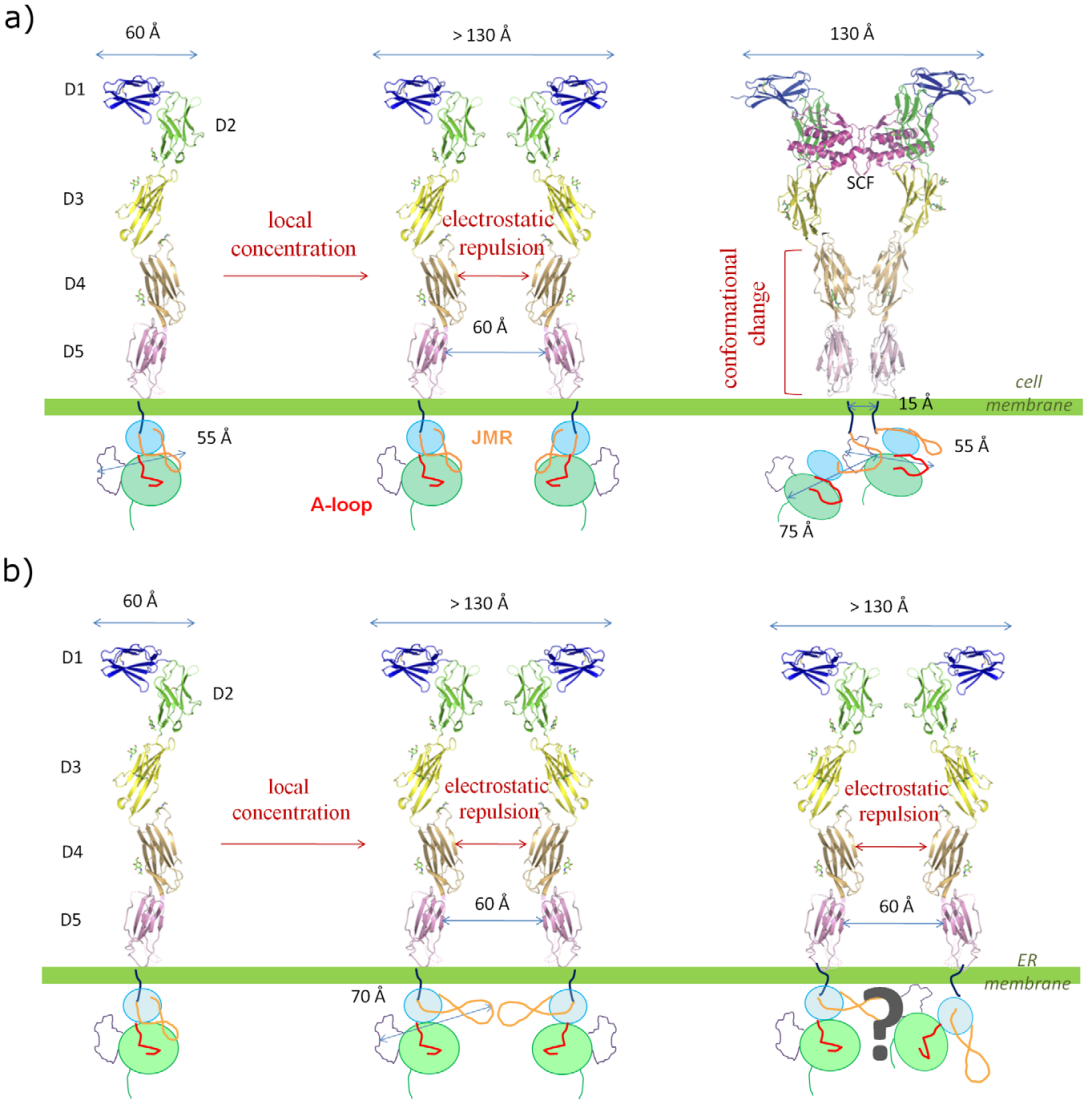
Schematic representation of a proposed model for wild-type and D816-mutated KIT dimerization and activation processes. (**a**) In wild-type KIT, the activation process supposedly unfolds as follows: (1) the receptor is anchored at the cell membrane in form of a monomer (PDB code 3EC8), (2) the density increases, but a minimal distance of 60 Å is kept between monomers due to electrostatic repulsion between domains D4 (mechanism proposed in [15]), (3) binding of SCF dimeric ligand promotes dimerization of KIT ectodomain and induces a conformational twist that brings the domains D5 of the two monomers in contact, (4) transmembrane helices of the two monomers are as close as 15 Å to each other, permitting dimerization sans transphosphorylation of KIT cytoplasmic region. (**b**) The D816V mutant is activated and triggers signaling pathways when anchored to the Endoplasmic Reticulum (ER) membrane, so that it does not meet the extra-cellular SCF ligand (mechanism proposed in [43]). Electrostatic repulsion between domains D4 of KIT monomers still holds, preventing dimerization and conformational twist of the ectodomain. However, our results show that JMR is allowed more freedom of movement and can spontaneously extend from PTK. Based on our NMA-displaced conformations, we can evaluate at about 20 Å at least the increase in the longest dimension of the protein. This increase could be sufficient to overcome the gap imposed by D4-D4 electrostatic repulsion and promote spontaneous dimerization of KIT cytoplasmic region, explaining why the D816V mutant functions as a dimer. The model of mutant dimer was drawn based on the lowest-energy complex predicted by RosettaDock (communicated in [115]) using KIT 4-Å conformation displaced along normal mode 17_{2531-ps}_.

Another topic can be considered in the discussion: the question of whether dimerization is mandatory for D816V mutant activity. Kanakura et al. proposed that the D814V mutant of murine KIT (equivalent to D816V mutant of human KIT) acts as a dimer, the dimerization interface is not located in the ectodomain and the last exerts only negative regulation on the ligand-independent dimerization process [44]. Later, structural studies of KIT ectodomain illustrated this supposed negative regulation by revealing a large conformational change between monomeric and dimeric forms [14], [15]. Monomeric ectodomains encounter electrostatic repulsion through their domain D4, maintaining receptors at a minimum distance from each other. Upon SCF binding, domains D4 and D5 twist and form a contacting interface (**Figure 9a**). Recently, Bougherara et al. showed that D816V mutant was able to induce downstream oncogenic signaling without the need to reach the cell surface [43]. Our results shed new light on these experimental data. Our description of the molecular mechanism by which the activating D816V mutation promotes spontaneous detachment of JMR from PTK, the triggering first step of the enzyme inactive-to-active state transition, may reconcile the views of a functioning dimeric mutant, yet a mutant activated without the need for extra-cellular ligand binding. Indeed, the greater freedom of movement of JMR in the mutant implies an increased longest dimension of KIT cytoplasmic region, suggesting that JMR could act as an arm able to extend from PTK toward other interacting partners such as another KIT kinase monomer (**Figure 9b**). Under such hypothesis, dimerization and transphosphorylation of KIT kinase would still hold as the activation mechanism of the mutated enzyme. Recent biochemical and structural characterization of RTK dimers has shown a great variety of interfaces [81]. It was suggested that FGFR1 – another receptor tyrosine kinase – ectodomain dimer formation imposes steric constraints that reduce the number of possible interaction modes between kinase domains [82]. As a consequence, loss-of-function mutation located in the interface prevents *in vivo* activation of the receptor. In a reciprocal manner in KIT, according to the model we propose, the gain-of-function mutation D816V could permit the kinase domains to bypass the repulsion between ectodomains in the absence of ligand, allowing for receptor activation.

This study proposes atomic level description of the regulatory impact of the D816V mutation, through local and remote structural/dynamic changes. The conformational exploration of the kinases – particularly the receptor tyrosine kinase KIT - specific inactive states presents an obvious therapeutic interest. Understanding of the regulation/deregulation of kinase activation contributes to the design of novel generation of inhibitors targeting KIT and other structurally or functionally related kinases. As an illustration, the efficiency of Gleevec™ to treat chronic myelogenous leukemia (CML) and gastrointestinal stromal tumors (GIST) is a consequence of its capacity to bind to and stabilize the inactive form of KIT and its binding preferences are governed by conformational selection [83]. The multi-approach procedure we applied on KIT inactive structure enabled us to postulate a model where the mutated kinase is able to dimerize without the enlisting of the extra-cellular domain. Very recent unpublished data (Schlessinger, personal communication, 2010) suggest that two populations of wild-type KIT dimers coexist in the cell, both showing a symmetric arrangement of the extra-cellular domain but an asymmetric arrangement of the kinase domain. Those data could be used in the future to construct reliable models for the homo (mutant/mutant) or hetero (wild-type/mutant) dimers of KIT receptor. The extensive NMA study of both wild-type and mutant KIT we conducted and the careful selection of a set of relevant modes could be further exploited to determine a plausible conformational transition pathway between the inactive and active states. The pocket profiles we obtained also encourages us to search for putative allosteric binding sites that could be targeted by small molecules that would trap the enzyme in an active conformation. Compared to orthosteric sites, allosteric sites are less-well conserved and thus ligands acting at allosteric sites have a greater potential to achieve receptor selectivity.

## Materials and Methods

### Bioinformatics analysis of X-ray structures

The crystallographic structures representing the auto-inhibited inactive and active states of KIT cytoplasmic region (PDB entries: 1T45 [69] and 1PKG [40]) were analyzed using the bioinformatics tools DSSP [84] and Stride [85]. (i) The secondary structure elements were assigned with the two algorithms, based either on the inter-molecular H-bonds (DSSP) or on both backbone geometry and inter-molecular H-bonds (Stride). (ii) Hydrogen bond networks (interactions D–H•••A, where D is the H-donor atom, A is the H-acceptor atom) were characterized with HBPLUS 3.2 [86] and visualized with PyMOL 1.2 [87] and Maestro (Schrödinger LLC, New York NY). The H-bond assignment was made using geometrical criteria [88], [89].

### Molecular dynamics simulations

#### Preparation of initial coordinate files

For the MD simulations of inactive KIT cytoplasmic region (CR), we used the crystallographic coordinates file of the auto-inhibited inactive form of KIT (1T45, 1.90 Å resolution) [69] retrieved from the PDB [90]. All crystallographic water molecules and other non-protein molecules were removed. Four initial models were generated: (I) wild-type full-length CR, WT^547-935^; (II) D816V-mutated full-length CR, MU^547-935^; (III) wild-type truncated CR, WT^567-935^ and (IV) D816V-mutated truncated CR, MU^567-935^. The truncated models were built by removing the N-terminal residues 547 to 566, part of the juxta-membrane region (JMR).

For equilibration of active KIT CR, we used the crystallographic coordinates file 1PKG (2.90 Å resolution) [40]. Chain A was retained, missing parts in the KID (residues 694 and 753–762) and in the C-tail (residues 927–935) were modeled with MODELLER 9v7 [91], [92] using 1T45 as a template; phosphates were removed from Y568 and Y570 of JMR. *In silico* substitution of D816 into a valine (V) was performed with MODELLER 9v7 [91], [92]. After these modifications, the model of active wild-type KIT (respectively, mutant) and the truncated model of inactive wild-type KIT (respectively, mutant) were comparable starting constructs.

All six models were prepared using the LEAP module of AMBER 10 [93], [94], with the parm99 parameter set: (i) hydrogen atoms were added, (ii) Na^+^or Cl^−^ counterions were added to neutralize the systems charge, (iii) the solute was hydrated with a box of explicit TIP3P water molecules [95] with a buffering distance up to 12 Å. The details of structure preparation and solvent models are given in **Table S1**.

#### Set up of the systems

The set up of the systems was performed with the SANDER module of AMBER 10 [94]. A minimization procedure similar to that used in [55] was followed: (i) 10 000 steps of minimization of water molecules keeping protein atoms fixed, (ii) 10 000 steps of minimization keeping only protein backbone fixed to allow protein side chains to relax, (iii) 10 000 steps of minimization without any constraint on the system. Heating of the system to the target temperature of 310 K was performed at constant volume using the Berendsen thermostat [96] and while restraining the solute C_α_ atoms by 10 kcal/mol/Å^2^. Thereafter, the system was equilibrated for 100 ps at constant volume (NVT) and for further 100 ps using a Langevin piston [97] (NPT) to maintain the pressure. Finally the restraints were removed and the system was equilibrated for a final 100-ps run. Backbone deviations obtained after equilibration are reported in Table S1.

#### Production of the trajectories

Six NPT simulations – two for WT^547-935^, two for MU^547-935^, one for WT^567-935^ and one for MU^567-935^ – were run during 50 ns on the equilibrated structures using the PMEMD module of AMBER 10. The temperature was kept at 310 K and pressure at 1 bar using the Langevin piston coupling algorithm. The SHAKE algorithm was used to freeze bonds involving hydrogen atoms, allowing for an integration time step of 2.0 fs. The Particle Mesh Ewald method (PME) [98] was employed to treat long-range electrostatics.

#### Analysis of the trajectories

Unless otherwise stated, MD trajectories were analyzed with the PTRAJ module of AMBER 10. From the root mean square deviation (RMSD) profiles, 2 ns were found sufficient for the systems to relax and the last 48 ns were retained for further analyses.

A convergence analysis was performed on the MD trajectories using an ensemble-based approach developed by Lyman & Zuckermann [71]. The algorithm makes use of the global C_α_ RMSD to discriminate representative MD conformations. The procedure for each trajectory can be described as follows: (i) a set of reference structures are identified, (ii) the MD conformational ensemble is clustered into corresponding reference groups. Each reference structure is first picked up at random and associated with a bin of conformations distant by less than an arbitrary cutoff *r*. Then the trajectory is split in two halves and conformations from each half are grouped based on their RMSD from each reference structure. A good convergence quality is assessed when each reference group is populated by conformations from the two halves of the trajectory at equivalent levels, meaning that every reference structure is equivalently represented in both halves of the trajectory.

The secondary structure assignment tool within PTRAJ makes use of DSSP [84]. Solvent accessible surface areas were computed with MSMS [99], using a probe sphere radius of 1.5 Å. Interactions were calculated by the HBPLUS 3.2 program in the LIGPLOT 4.5.3 package [100]. VMD 1.8.7 [101] and PyMOL 1.2 were used for visualization and the analysis graphics were drawn using the R package [102].

### Protein energetics analysis

Free energies were evaluated using the Molecular Mechanism Generalized Born Surface Area (MMGBSA) method – implemented in AMBER 10 [73], [103]–[105] which proposes to express the total free energy of the protein as a sum of contributions
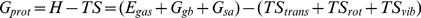
(1)where *H* is the enthalpy and *TS* is the configurational entropy of the solute. *E_gas_* is the molecular mechanics energy of the solute, obtained by summing the internal energy *E_int_*, the electrostatics interactions *E_ele_* and the van-der-Waals contacts *E_vdw_*; *G_gb_* is the polar solvation term whose evaluation is based on the continuum generalized Born solvent model; *G_sa_* is the non-polar solvation term, proportional to the solvent accessible surface area (SASA), and was evaluated using the Linear Combinations of Pairwise Overlaps (LCPO) method. The translational *TS_trans_*, rotational *TS_rot_* and vibrational *TS_vib_* entropies, were evaluated through normal mode calculations with the NMODE module, using a dielectric constant of 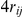, where 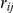 is the distance between atoms *i* and *j*.

In principle, considering a two-state model, the calculation of the free energy difference between the wild-type and the mutant inactive-folded and unfolded states would be required to evaluate the protein stability changes. Following the assumption that, in the unfolded state, individual residues may not interact and hence the contributions of all the residues except the one under mutation (D816V) are the same in the wild-type and the mutant, we considered that this difference between one-residue contributions should be small compared to the difference between the total energies of the wild-type and mutated folded states. We thus evaluated the thermodynamic stability difference for the inactive state directly from our MD simulations of WT^547-935^ and MU^547-935^, as:
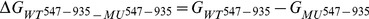
(2)


The quantities 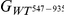 and 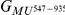 were averaged over 2400 snapshots selected at 20-ps intervals along the four 50-ns MD simulations and the protein stability change was then approximated based on the free energy difference 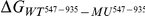. Statistical errors on estimates were calculated from their variance and auto-correlation function using the method of Straatsma [74]:
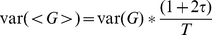
(3)where var(*G*) is the variance of the estimate, 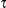 is the correlation length from the relaxation of the autocorrelation, and 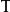 is the total length of the time series.

The relative free energies of active versus inactive equilibrated conformations were also evaluated for wild-type and mutant KIT truncated CR (single-point calculations).

The free energy of binding a ligand to a receptor is defined as:
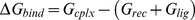
(4)


Here we computed the binding free energy of JMR and its fragments, the remaining parts of the protein being considered as the receptor, to get estimates of the different energetic contributions involved in the attachment of JMR to PTK. The binding free energies were evaluated on the equilibrated conformations of WT^547-935^ and MU^547-935^ (single-point calculations). The change in JMR (or its fragments) relative stability within the protein was then approximated based on the difference between the binding energies 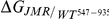 and 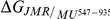.
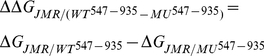
(5)


### Normal modes analysis

Normal mode analyses (NMA) were conducted using the DIMB method [106] of the VIBRAN module of CHARMM 35b3 [107], [108] on (i) the crystallographic structure 1T45 [69], (ii) MD conformations from WT^547-935^ taken at 4217, 34238, 42356, 49260 ps, (iii) MD conformations from MU^547-935^ taken at 2531, 18157, 30180, 36987 ps. The selected MD conformations were found to be the most representative of the trajectories, according to the convergence analysis. The first hydration shell (<5 Å, 2200 water molecules) around the MD conformations was kept to help prevent the solvent-exposed regions of the protein from collapsing during the minimization procedure [78]. During initial steepest descent energy minimization of the system, mass-weighted harmonic constraints (250 kcal/mol/A^2^) were applied to the starting structure and reduced by a factor of 2 every 1000 minimization steps until they fell below a threshold value of 5 kcal/mol/A^2^. The constraints were then removed and the system was minimized by conjugate gradient and adopted-basis Newton-Raphson steps until the RMS energy gradient fell below 10^−5^ kcal/mol/A. The C_α_ RMS deviations of the minimized conformations from their initial position were limited to less than 1 Å (**Table S2**). Normal modes were computed by diagonalizing the mass-weighted Hessian matrix of the energy-minimized conformations and the 97 non-zero lowest-frequency modes were analyzed.

The degree of collectivity of the JMR motions in a given mode *l* was calculated as [109], [110]:
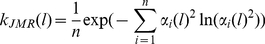
(6)where *n* = 596 is the number of atoms belonging to JMR. The quantity 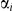 is defined as:
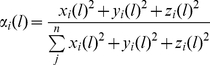
(7)where 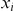, 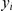 and 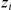 are the components of mode *l* that correspond to the three degrees of freedom of atom *i* and such that 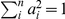. The degree of collectivity is comprised between 0 and 1. A value of 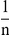 indicates that only one atom is involved in the motion while a value close to 1 indicates high collectivity.

The resultant displacement, i.e. the norm of the resultant displacement vector, of any fragment of the protein was calculated as:
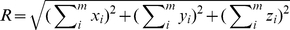
(8)over the ensemble *M* of the *m* atoms belonging to the fragment – 172 for JM-Switch and 181 for JM-Zipper.

Displacements along selected normal modes, in both positive and negative directions, were performed with the VMOD facility in CHARMM 35b3. The range of displacement was set from −4 Å to 4 Å with steps of 0.1 Å with respect to the initial conformations extracted from WT^547-935^ and MU^547-935^ MD trajectories. Intermediate conformations were obtained using a restraint potential added to the internal standard potential [111], [112].

Pockets were detected at the surface of the crystallographic structures 1T45 and 1PKG and the 4-Å displaced conformations using fpocket [113], with the default parameters. This geometry-based algorithm was found to perform best on accurate binding site prediction in a recent large-scale comparison study [114]. Pockets were selected by visual inspection with PyMOL 1.2.

## Supporting Information

Figure S1 MD simulations of KIT cytoplasmic region with cleaved JMR in the inactive form. The RMS deviations (in Å) were calculated from MD simulations of truncated wild-type Kit, WT^567–935^ (in green), and D816V mutant, MU^567–935^ (in purple), on the backbone atoms of: (**a**) the whole protein, (**b**) N-lobe, (**c**) C-lobe, (**d**) A-loop and (**e**) JMR, with respect to the initial frame. The dashed grey vertical line drawn at 2 ns indicates the relaxation time.
(TIFF)

Figure S2 Relative free energies of active versus inactive states of wild-type and D816V-mutated KIT truncated CR. Free energies were computed on the equilibrated conformations of WT^567–935^ and MU^567–935^ in the inactive (I) and active (A) states.
(TIFF)

Figure S3 Superimposition of KIT inactive 50-ns MD conformations and active X-ray structure. The 50-ns final MD conformations of WT^547–935^ (in opaque and transparent dark blue), of MU^547–935^ (in opaque and transparent red), of WT^567–935 ^(in green) and of MU^567–935 ^(in purple), and the X-ray structure 1PKG (chain B, in transparent black) were superimposed. (**A**) On the left are displayed the JMR (residues 553 to 581 for 1PKG,WT^547–935^ and MU^547–935^, residues 567 to 581 for WT^567–935^ and MU^567–935^), helix C and its preceeding loop (residues 627 to 648) and the A-loop (residues 810 to 839), in cartoon representation; on the right is displayed a zoom of the 820–839 part of the A-loop and an helix from the C-lobe that serves as a platform for the substrate in 1PKG (residues 874 to 887). (**B**) The table in insert gives the atomic fluctuations mean, maximum and median values in Å2 for JMR.
(TIFF)

Figure S4 Degrees of collectivity of JMR atomic motions. Normal mode analysis was performed on (i) the crystallographic structure 1T45 of wild-type KIT cytoplasmic region, (ii) four conformations representative of WT^547–935^ MD trajectory **1**, (iii) four conformations representative of MU^547–935^ MD trajectory **1**. The degrees of collectivity *k_JMR_* were computed on the 97 lowest-frequency modes obtained from each NMA, leading to a total of 97 values for the X-ray structure and 388 values forWT^547–935^ and MU^547–935^ respectively. The histograms give the distributions of the 388 *k_JMR_* values for the wild type (in blue) and the mutant (in red). The dotted lines indicate the corresponding mean *k_JMR_* values and the mean *k_JMR_* value for the X-ray structure.
(TIFF)

Figure S5 Total energies and MMFP2 restraints recorded along the 4-Å displacement of wild-type and D816V mutated KIT cytoplasmic domain structure along chosen normal modes. **Upper Panel:** the wild-type, WT^547–935^. **Lower Panel:** the mutant D816V, MU^547–935^. Plain and dashed lines give the total energy and the MMFP2 restraint respectively, in kcal/mol. The red color indicates the direction followed to the extreme conformations represented on Figure 8, which are labeled as red crosses.
(TIFF)

Figure S6 Pockets detected on the surface of wild-type and D816V-mutated KIT receptor truncated cytoplasmic region. **Left Panel:** the wild-type, WT^567–935^. **Right Panel:** the mutant D816V, MU^567–935^. Conformations were extracted after 50 ns of MD simulation. The core of the protein is in cyan, JMR is in orange and A-loop is in red. Pockets are displayed in red when they overlap with the catalytic site, in orange when they are located between the catalytic site and JMR, in olive, green or purple when they overlap with the substrate binding site.
(TIFF)

Table S1 MD preparation and equilibration details. The counter-ions employed to neutralize the systems are Na^+^ and Cl^−^. Root mean square deviations were computed on the backbone atoms of the equilibrated conformations versus the initial template.
(PDF)

Table S2 RMS deviations (in Å) upon energy minimization calculated on C-α atoms for every MD conformation used for NMA. The value obtained for the structure 1T45 is 1.55 Å.
(PDF)
