# Supplementary figures and images for: Mutation D816V Alters the Internal Structure and Dynamics of c-KIT Receptor Cytoplasmic Region: Implications for Dimerization and Activation Mechanisms
Source: PLoS Comput Biol. 2011 Jun 16;7(6):e1002068. doi: 10.1371/journal.pcbi.1002068 (PMC3116893; doi:10.1371/journal.pcbi.1002068)

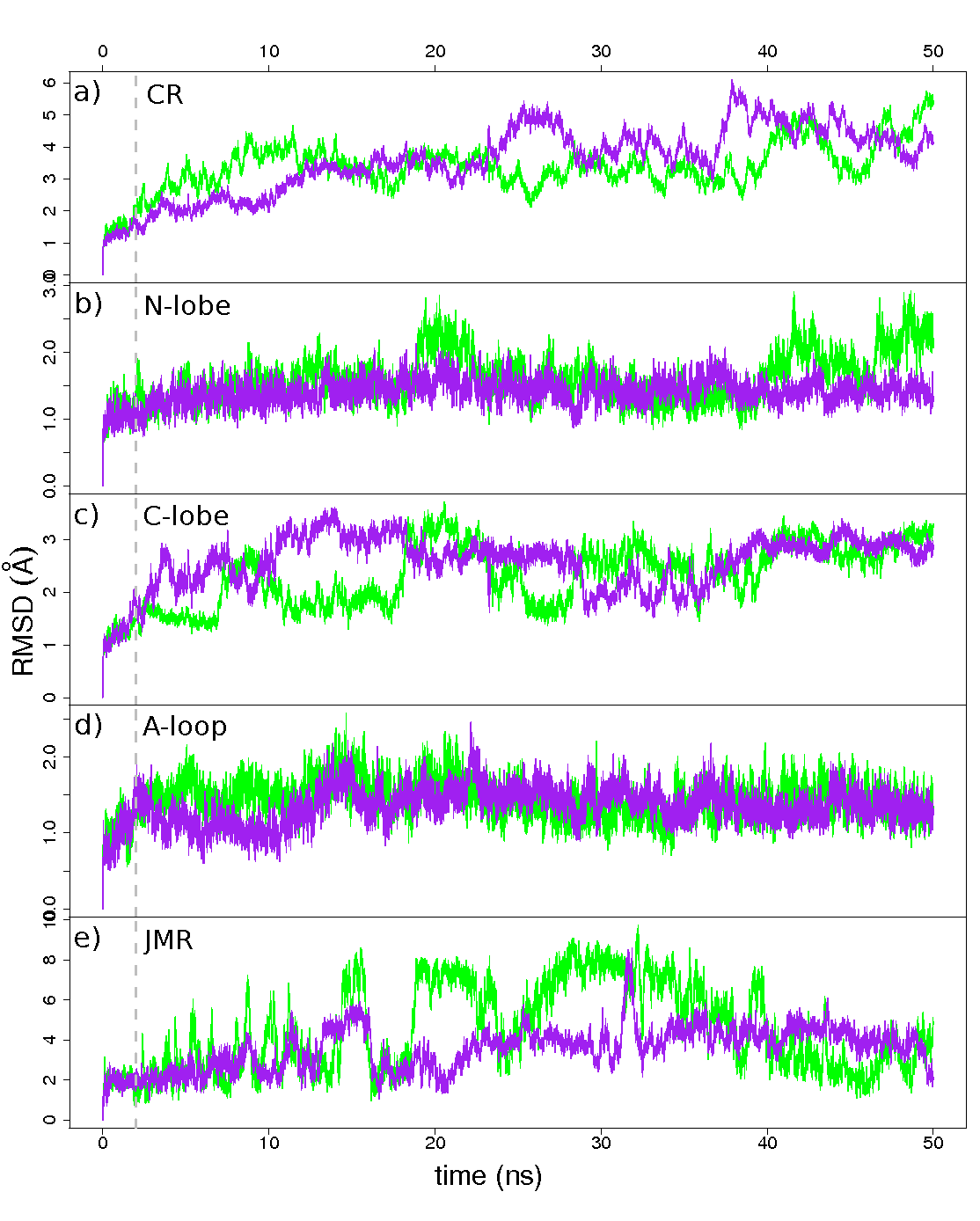

Supplement: Figure S1 — MD simulations of KIT cytoplasmic region with cleaved JMR in the inactive form. The RMS deviations (in Å) were calculated from MD simulations of truncated wild-type Kit, WT567–935 (in green), and D816V mutant, MU567–935 (in purple), on the backbone atoms of: (a) the whole protein, (b) N-lobe, (c) C-lobe, (d) A-loop and (e) JMR, with respect to the initial frame. The dashed grey vertical line drawn at 2 ns indicates the relaxation time. (TIFF) [file pcbi.1002068.s001.tiff]

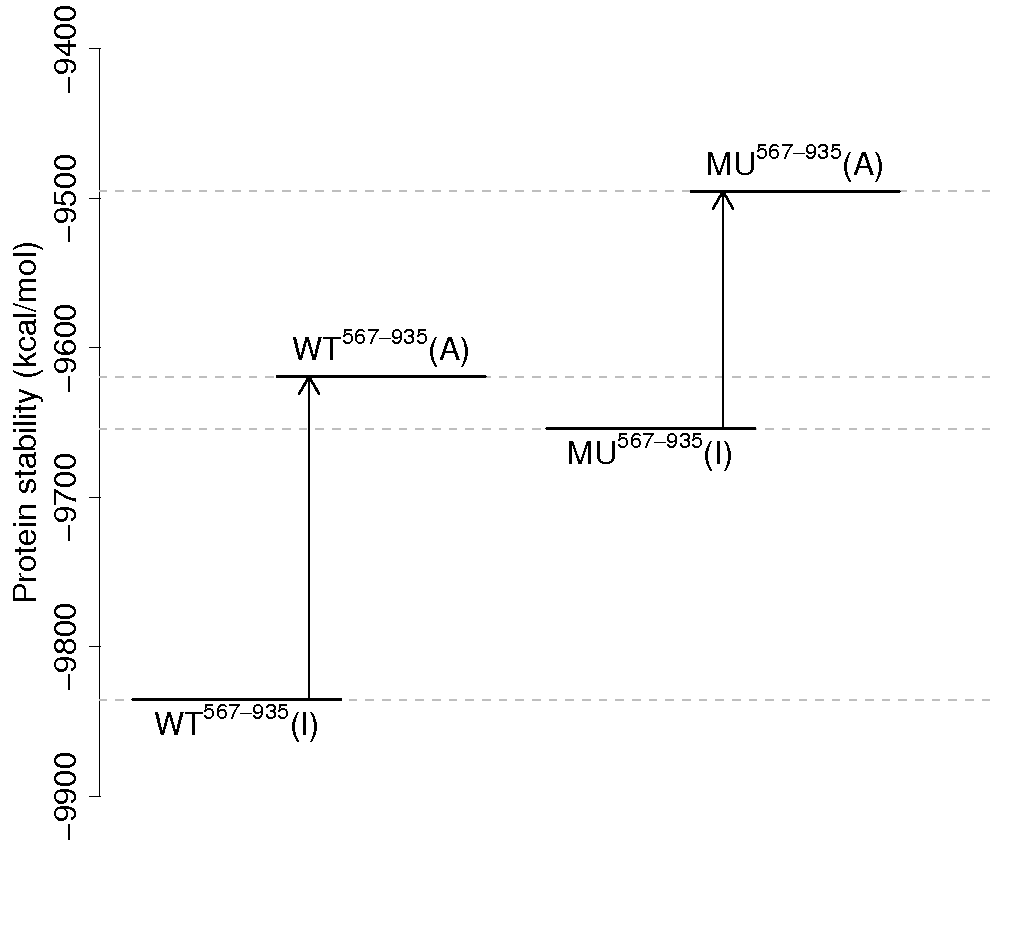

Supplement: Figure S2 — Relative free energies of active versus inactive states of wild-type and D816V-mutated KIT truncated CR. Free energies were computed on the equilibrated conformations of WT567–935 and MU567–935 in the inactive (I) and active (A) states. (TIFF) [file pcbi.1002068.s002.tiff]

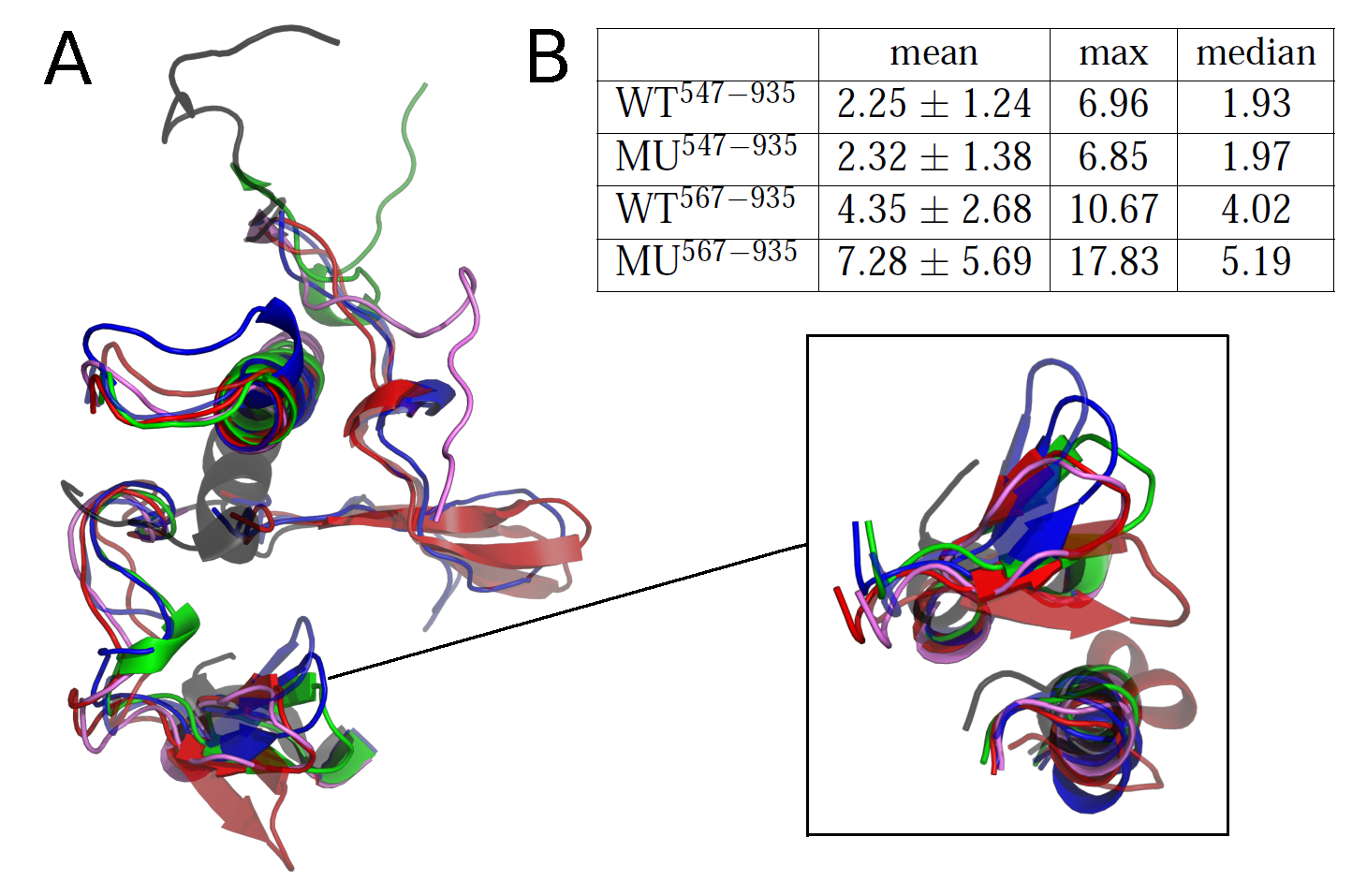

Supplement: Figure S3 — Superimposition of KIT inactive 50-ns MD conformations and active X-ray structure. The 50-ns final MD conformations of WT547–935 (in opaque and transparent dark blue), of MU547–935 (in opaque and transparent red), of WT567–935 (in green) and of MU567–935 (in purple), and the X-ray structure 1PKG (chain B, in transparent black) were superimposed. (A) On the left are displayed the JMR (residues 553 to 581 for 1PKG,WT547–935 and MU547–935, residues 567 to 581 for WT567–935 and MU567–935), helix C and its preceeding loop (residues 627 to 648) and the A-loop (residues 810 to 839), in cartoon representation; on the right is displayed a zoom of the 820–839 part of the A-loop and an helix from the C-lobe that serves as a platform for the substrate in 1PKG (residues 874 to 887). (B) The table in insert gives the atomic fluctuations mean, maximum and median values in Å2 for JMR. (TIFF) [file pcbi.1002068.s003.tiff]

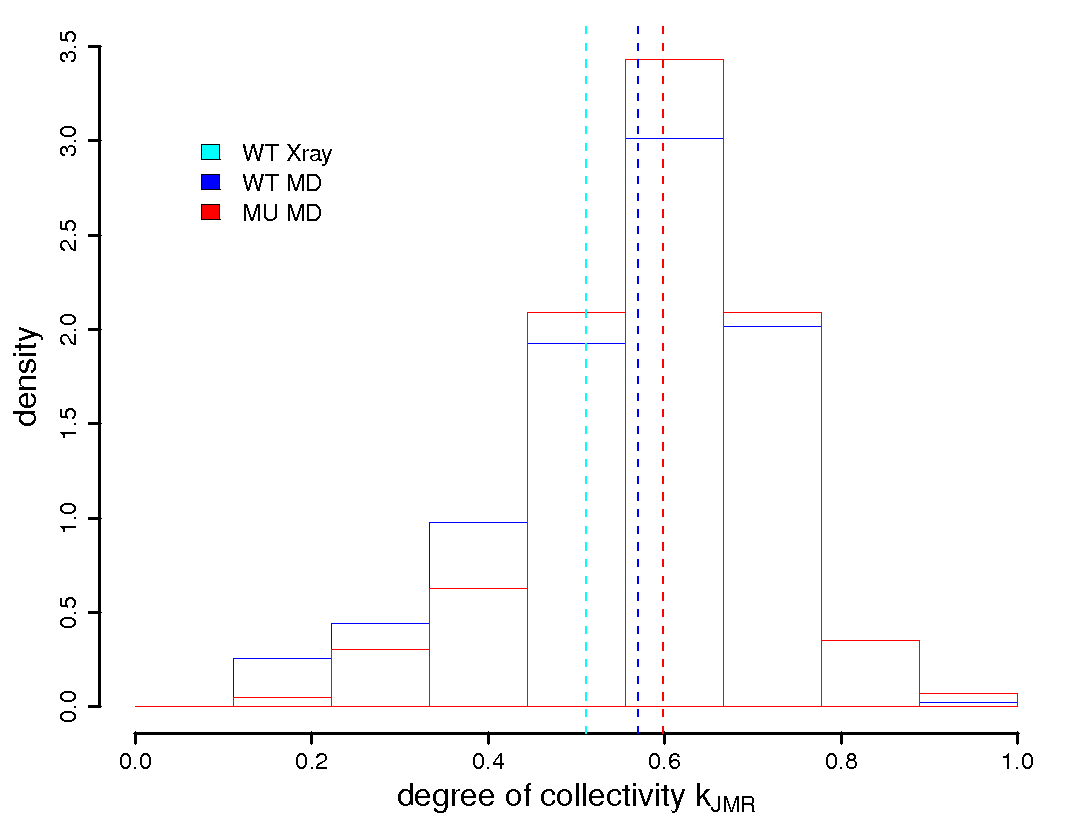

Supplement: Figure S4 — Degrees of collectivity of JMR atomic motions. Normal mode analysis was performed on (i) the crystallographic structure 1T45 of wild-type KIT cytoplasmic region, (ii) four conformations representative of WT547–935 MD trajectory 1, (iii) four conformations representative of MU547–935 MD trajectory 1. The degrees of collectivity kJMR were computed on the 97 lowest-frequency modes obtained from each NMA, leading to a total of 97 values for the X-ray structure and 388 values forWT547–935 and MU547–935 respectively. The histograms give the distributions of the 388 kJMR values for the wild type (in blue) and the mutant (in red). The dotted lines indicate the corresponding mean kJMR values and the mean kJMR value for the X-ray structure. (TIFF) [file pcbi.1002068.s004.tiff]

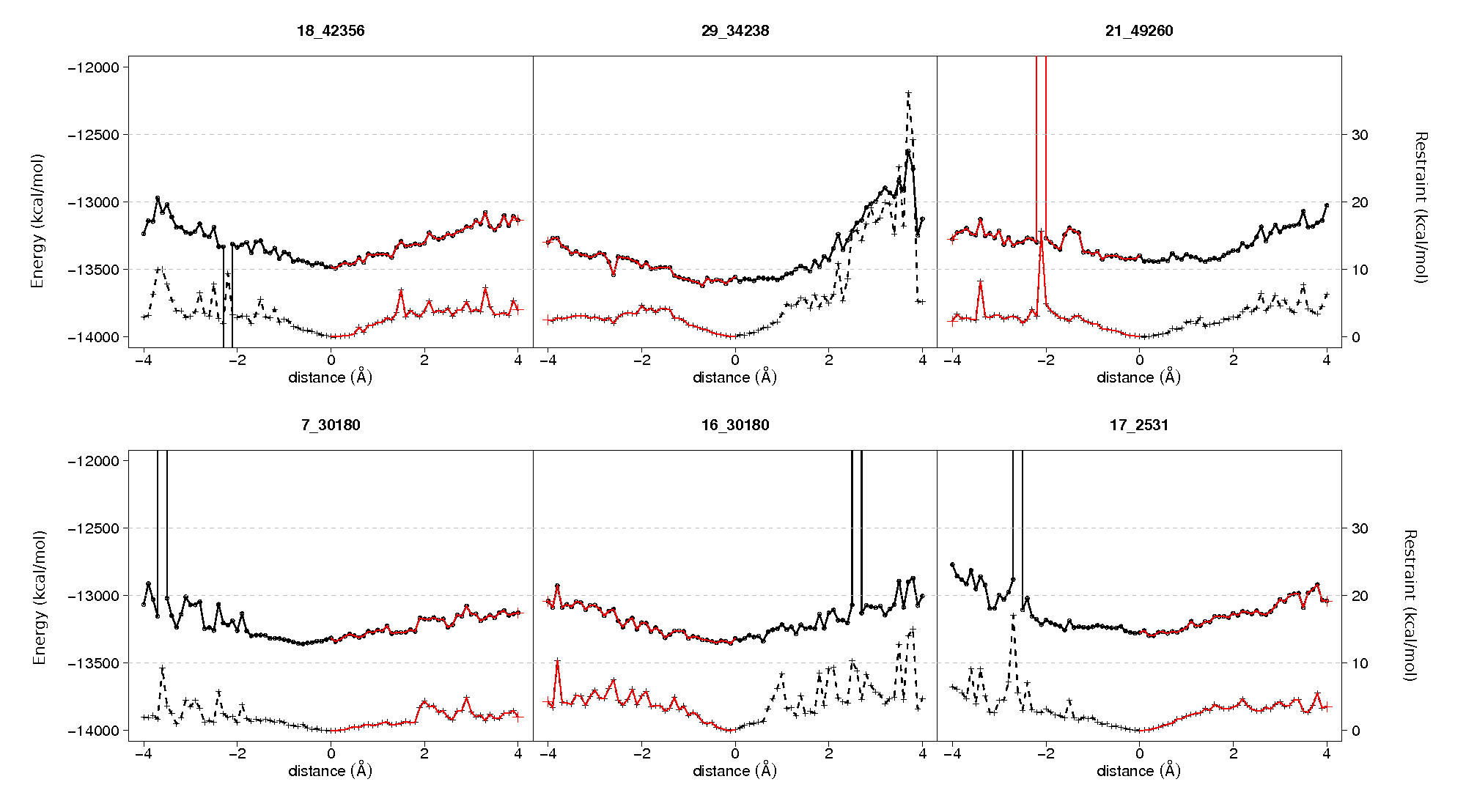

Supplement: Figure S5 — Total energies and MMFP2 restraints recorded along the 4-Å displacement of wild-type and D816V mutated KIT cytoplasmic domain structure along chosen normal modes. Upper Panel: the wild-type, WT547–935. Lower Panel: the mutant D816V, MU547–935. Plain and dashed lines give the total energy and the MMFP2 restraint respectively, in kcal/mol. The red color indicates the direction followed to the extreme conformations represented on Figure 8, which are labeled as red crosses. (TIFF) [file pcbi.1002068.s005.tiff]

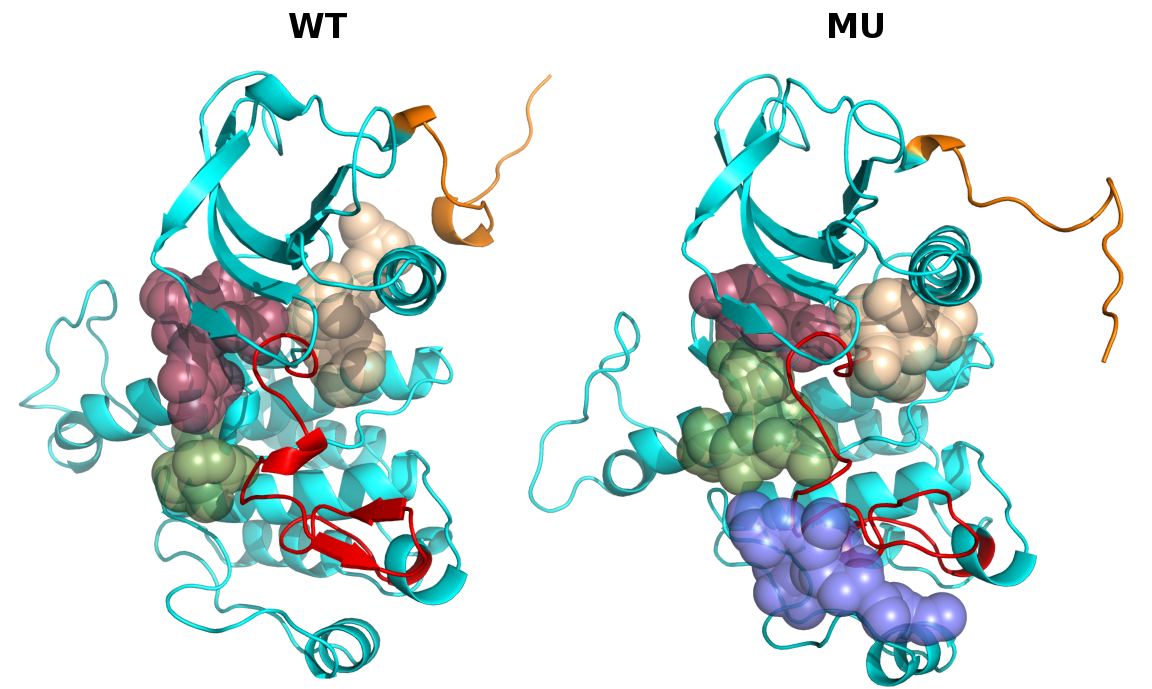

Supplement: Figure S6 — Pockets detected on the surface of wild-type and D816V-mutated KIT receptor truncated cytoplasmic region. Left Panel: the wild-type, WT567–935. Right Panel: the mutant D816V, MU567–935. Conformations were extracted after 50 ns of MD simulation. The core of the protein is in cyan, JMR is in orange and A-loop is in red. Pockets are displayed in red when they overlap with the catalytic site, in orange when they are located between the catalytic site and JMR, in olive, green or purple when they overlap with the substrate binding site. (TIFF) [file pcbi.1002068.s006.tiff]
