# Supplementary material for: Mutation D816V Alters the Internal Structure and Dynamics of c-KIT Receptor Cytoplasmic Region: Implications for Dimerization and Activation Mechanisms
Source: PLoS Comput Biol. 2011 Jun 16;7(6):e1002068. doi: 10.1371/journal.pcbi.1002068 (PMC3116893; doi:10.1371/journal.pcbi.1002068)
Supplement: Table S1 — MD preparation and equilibration details. The counter-ions employed to neutralize the systems are Na+ and Cl−. Root mean square deviations were computed on the backbone atoms of the equilibrated conformations versus the initial template. (PDF) [file pcbi.1002068.s007.pdf]

	auto-inhibited inactive state		truncated inactive state		active state	
	$WT^{547-935}$	$MU^{547-935}$	$WT^{567-935}$(I)	$MU^{567-935}$(I)	$WT^{567-935}$(A)	$MU^{567-935}$(A)
Total charge of counter-ions	+1	0	0	-1	0	-1
Water box dimensions (Å^3)	76.7 x 72.8 x 80.9		83.1 x 76.5 x 82.9		84.2 x 71.8 x 84.4	
Number of water molecules	13 195	13 197	15 739	15 733	12 003	11 997
Total number of atoms	44 870	44 879	52 160	52 147	40 952	40 939
Deviation after equilibration (Å)	1.29	1.13	1.22	1.24	1.82	1.40

Table S1. MD preparation and equilibration details. The counter-ions employed to neutralize the systems are Na^+ and Cl^-. Root mean square deviations were computed on the backbone atoms of the equilibrated conformations versus the initial template.
