# Supplementary material for: Mutation D816V Alters the Internal Structure and Dynamics of c-KIT Receptor Cytoplasmic Region: Implications for Dimerization and Activation Mechanisms
Source: PLoS Comput Biol. 2011 Jun 16;7(6):e1002068. doi: 10.1371/journal.pcbi.1002068 (PMC3116893; doi:10.1371/journal.pcbi.1002068)
Supplement: Table S2 — RMS deviations (in Å) upon energy minimization calculated on C-α atoms for every MD conformation used for NMA. The value obtained for the structure 1T45 is 1.55 Å. (PDF) [file pcbi.1002068.s008.pdf]

| $WT^{547-935}$ | | $MU^{547-935}$ | |
|---|---|---|---|
| conformation | rms | conformation | rms |
| 4217 | 0.65 | 2531 | 0.68 |
| 34238 | 0.68 | 19157 | 0.67 |
| 42356 | 0.64 | 30180 | 0.65 |
| 49260 | 0.58 | 36987 | 0.73 |

**Table S2. RMS deviations (in Å) upon energy minimization calculated on C-α atoms for every MD conformation used for NMA.** The value obtained for the structure 1T45 is 1.55 Å.
